# *Mafb* and *c-Maf* Have Prenatal Compensatory and Postnatal Antagonistic Roles in Cortical Interneuron Fate and Function

**DOI:** 10.1016/j.celrep.2019.01.031

**Published:** 2019-01-29

**Authors:** Emily Ling-Lin Pai, Daniel Vogt, Alexandra Clemente-Perez, Gabriel L. McKinsey, Frances S. Oho, Jia Sheng Hu, Matt Wimer, Anirban Paul, Siavash Fazel Darbandi, Ramon Pla, Tomasz J. Nowakowski, Lisa V. Goodrich, Jeanne T. Paz, John L.R. Rubenstein

**Affiliations:** 1 Nina Ireland Laboratory of Developmental Neurobiology, Department of Psychiatry, UCSF Weill Institute for Neurosciences, University of California, San Francisco, San Francisco, CA 94158, USA; 2 Department of Pediatrics and Human Development, Michigan State University, Grand Rapids, MI 49503, USA; 3 Neuroscience Graduate Program, University of California, San Francisco, San Francisco, CA 94158, USA; 4 Department of Neurology, University of California, San Francisco, San Francisco, CA 94158, USA; 5 Gladstone Institute of Neurological Disease, San Francisco, CA 94158, USA; 6 Cold Spring Harbor Laboratory, Cold Spring Harbor, NY 11724, USA; 7 Department of Anatomy, Department of Psychiatry, University of California, San Francisco, San Francisco, CA 94158, USA; 8 Department of Neurobiology, Harvard Medical School, Boston, MA 02115, USA; 9 Present address: IDINE, Departamento de Ciencias Médicas, Facultad de Medicina, Universidad Castilla-La Mancha, 02006 Albacete, Spain; 10 Present address: College of Medicine, Penn State University, Hershey, PA 17033, USA; 11 These authors contributed equally; 12 Lead Contact

## Abstract

*Mafb* and *c-Maf* transcription factor (TF) expression is enriched in medial ganglionic eminence (MGE) lineages, beginning in late-secondary progenitors and continuing into mature parvalbumin (PV^+^) and somatostatin (SST^+^) interneurons. However, the functions of Maf TFs in MGE development remain to be elucidated. Herein, *Mafb* and *c-Maf* were conditionally deleted, alone and together, in the MGE and its lineages. Analyses of *Maf* mutant mice revealed redundant functions of *Mafb* and *c-Maf* in secondary MGE progenitors, where they repress the generation of SST^+^ cortical and hippocampal interneurons. By contrast, *Mafb* and *c-Maf* have distinct roles in postnatal cortical interneuron (CIN) morphological maturation, synaptogenesis, and cortical circuit integration. Thus, *Mafb* and *c-Maf* have redundant and opposing functions at different steps in CIN development.

## In Brief

Using mouse genetics and *ex vivo* physiology studies, Pai et al. show that *Mafb* and *c-Maf* together are necessary to generate the proper numbers of parvalbumin and somatostatin GABAergic interneurons. However, in maturing interneurons, *Mafb* and *c-Maf* function divergently to control their firing properties and communication with other neurons.

## Graphical Abstract

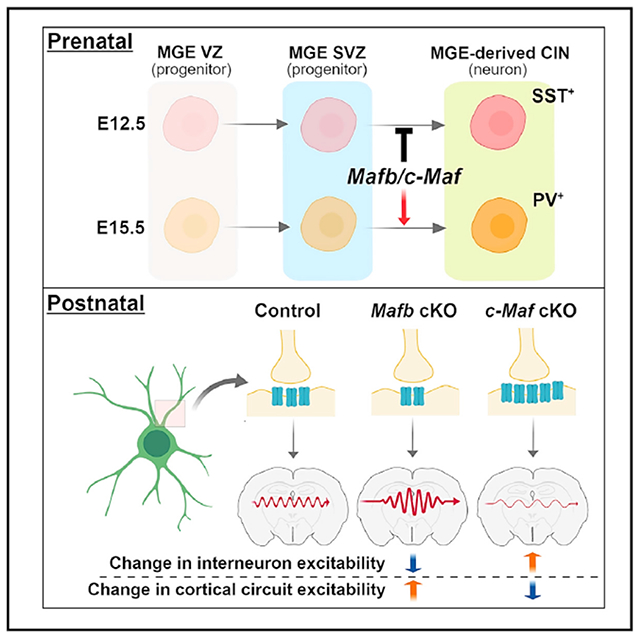

## INTRODUCTION

Disturbances in cortical development and maturation are thought to underlie some symptoms of neurological and neuropsychiatric disorders, such as autism spectrum disorder (ASD), epilepsy, and schizophrenia. One mechanism that is postulated to contribute to symptoms is a circuit imbalance in the excitation to inhibition (E/I) ratio (Chao et al., 2010; Han et al., 2012; Rubenstein and Merzenich, 2003; Yizhar et al., 2011). Although most cortical excitation is generated by glutamatergic projection neurons and thalamic afferents, inhibition is largely generated by locally projecting GABAergic cortical interneurons (CINs). CINs exhibit diverse morphological, connectivity, molecular, and electrophysiological properties (Huang et al., 2007; Kepecs and Fishell, 2014; Kessaris et al., 2014), which facilitate the E/I balance in distinct cortical microcircuits.

CINs are derived from progenitor zones in the subpallial telencephalon, called the medial and caudal ganglionic eminences (MGE and CGE, respectively), and the preoptic area (POA) (Gelman et al., 2011; Wonders and Anderson, 2006). The development of MGE and CGE-derived CINs are coordinated by a combination of transcription factors (TFs) expressed in these progenitor zones (Hu et al., 2017; Lim et al., 2018a). Mature CINs express molecular markers that delineate four broad subgroups: MGE-derived somatostatin (SST^+^) and parvalbumin (PV^+^), and CGE-derived vasoactive intestinal peptide (VIP^+^) and reelin^+^;SST^−^ (Lim et al., 2018a).

*Mafb* and *c-Maf*, a part of the large *Maf* TF family, bind to DNA through the basic leucine zipper motif (Kataoka, 2007). *Mafb* and *c-Maf* function alone or together to control cell fate and differentiation in bone, epithelial cells, lens, macrophages, and pancreas (Lopez-Pajares et al., 2015; Nishikawa et al., 2010; Soucie et al., 2016). In the nervous system, *Mafb* and *c-Maf* have multiple functions. For example, *Mafb* controls embryonic hindbrain regional patterning (Cordes and Barsh, 1994) and promotes the formation of auditory ribbon synapses that are required to activate inner hair cells (Lu et al., 2011; Yu et al., 2013). *c-Maf* is involved in touch receptor differentiation in the peripheral nervous system (Wende et al., 2012).

*Mafb* and *c-Maf* are particularly intriguing in CIN development because their MGE expression initiates in the MGE subventricular zone (SVZ) and persists in MGE-derived interneurons but not in MGE-derived projection neurons (Cobos et al., 2006; McKinsey et al., 2013; Zhao et al., 2008). In addition, TFs that control MGE CIN development also regulate *Mafb* and *c-Maf* expression. For instance, *Zfhx1b* mutants have reduced *c-Maf* expression, *Lhx6* mutants have reduced *Mafb* expression, and *Dlx1/2* mutants have reduced *Mafb* and *c-Maf* expression (McKinsey et al., 2013; Zhao et al., 2008). Two recent reports provided evidence that (1) *Mafb* is preferentially expressed in a subtype of SST^+^ CINs (Martinotti cells), where it regulates their migration and axonal projection (Lim et al., 2018b); and (2) *c-Maf* promotes the generation of SST^+^ CINs (Mi et al., 2018), a finding that is in opposition to the combined functions of *Mafb and c-Maf* presented herein.

Here, we report the individual and combined functions of *Mafb* and *c-Maf* after conditional deletion in the MGE lineages with *Nkx2.1-Cre* and *799-CreER. Mafb;c-Maf* conditional double knockout (cDKO) phenotypes provided evidence that *Mafb* and *c-Maf* compensate for each other. Notably, cDKOs generate excessive SST^+^ at the expense of PV^+^ CINs. Furthermore, cDKOs have reduced CIN numbers, probably because of a combination of mechanisms, including ectopic migration to the hippocampus and a progressive reduction in CINs during postnatal ages. However, our electrophysiological analyses of adult somatosensory cortices and *in vitro* assays of neonatal CINs provide evidence that *Mafb* and *c-Maf* have distinct postnatal functions in CIN maturation, synaptogenesis, and activity. Together these defects lead to alterations in neocortical circuit excitability and provide potential mechanistic insights into how these TFs operate during CIN development and maturation.

## RESULTS

### *Mafb* and *c-Maf* Have Overlapping and Distinct Expression Patterns in Developing CINs

**Figure 1.**
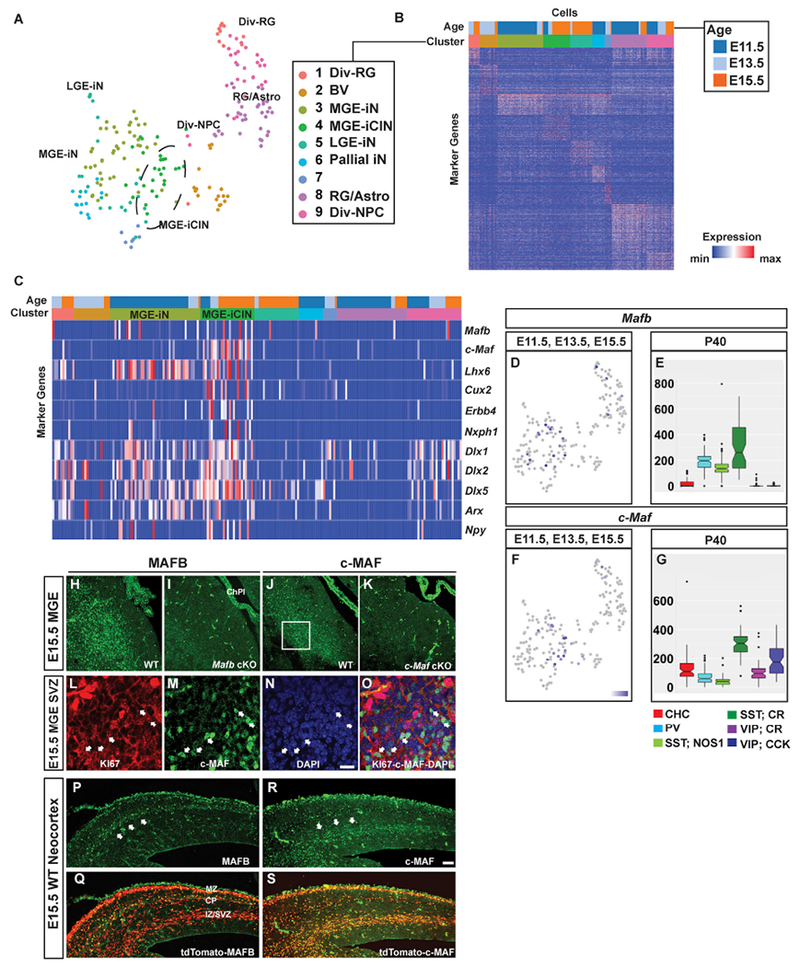
Analysis of *Mafb* and *c-Maf* Expression in Single Cells and in Tissue Sections from the MGE and in MGE-Derived Developing and Mature CINs (A–G) Single-cell RNA-seq MGE and adult CIN analyses. (A) t-stochastic neighbor embedding (t-SNE) plot representing MGE cells analyzed from age E11.5, E13.5, and E15.5 and colored by their cluster assignments. Cells with properties of MGE-derived immature CINs (iCIN) are encircled in cluster 4. (B) Heat map representation of cluster marker genes. Please see Table S1 for the gene list. (C) Heat map representation of the 9 clusters; it shows enrichment of *Mafb* and *c-Maf* in cluster 4, and the expression of markers for MGE-derived CINs. Notethat half of the cluster 4 CINs co-express *Mafb* and *c-Maf*. (D–G) Expression feature plot of *Mafb* (D) and *c-Maf* (F) in MGE cells; positive cells are blue. Box plots of *Mafb* (E) and *c-Maf* (G) expression in CIN subtypes at P40. (H and I) Immunofluorescent images of the MGE from E15.5 WT (H) and *Mafb* cKO (I) showing MAFB expression. (J and K) Immunofluorescent images of the MGE from E15.5 WT (J) and c-*Maf* cKO (K) showing c-MAF expression. (L–O) Confocal imaging that shows co-labeling of KI67 (L) and c-MAF (M) in the MGE SVZ (O) (boxed in J). Arrowheads point to cells that are c-MAF^+^ progenitors. (P–S) Immunofluorescent images from E15.5 WT neocortex that show *Nkx2.1-cre*-mediated tdTomato expression merged with MAFB (P–Q) and c-MAF (R–S). Scale barin (N) and (R) represents 100 μm. Div-RG, dividing radial glia; MGE-iN/iCIN, MGE-derived immature neurons/cortical interneurons; LGE-iN, LGE-derived immature neurons; pallial iN, pallial immature neurons; RG/Astro, radial glias and astrocytes; Div-NPC, dividing neural progenitor cells; CHC, chandelier cells; CR, calretinin; VIP, vasoactive intestinal peptide; ChPl, choroid plexus; MZ, marginal zone; CP, cortical plate; IZ/SVZ, intermediate zone-subventricular zone.

We compared *Mafb* and *c-Maf* gene expression in the MGE and at later stages of CIN maturation. First, we determined *Mafb* and *c-Maf* gene expression profiles and their cellular specificity in the MGE by reanalyzing single cell RNA sequencing (scRNA-seq) data derived from wild-type (WT) E11.5, E13.5, and E15.5 MGE tissue (Chen et al., 2017). We studied the data set by Louvain clustering with Jaccard distance matrix, which revealed nine molecularly distinct clusters (Figures 1A and 1B; Table S1) (Shekhar et al., 2016). *Mafb* and *c-Maf* were expressed in some MGE progenitor cells (clusters 1, 8, and 9; Table S1). In addition, *Mafb* and *c-Maf* mRNA were co-expressed in a subset of these progenitors. *Mafb and c-Maf* were significantly enriched in cluster 4, which we propose corresponds to cells that will become CINs, based on their expression of multiple genes, including *Cux2* and *Erbb4* (Figure 1C). The expression levels of *Mafb* and *c-Maf* in cluster 4 showed that these genes were largely co-expressed in immature CINs. Thus, *Mafb* and *c-Maf* expression initiates in progenitors, and their co-expression increases in immature CINs (Figures 1C, 1D, 1F, and 1H–1S).

We then assessed single-cell transcriptome data from postnatal day 40 (P40) CINs (Paul et al., 2017). These data showed that *Mafb* and *c-Maf* expression persisted in MGE-derived adult CINs. *Mafb* and *c-Maf* were enriched in multiple MGE-derived CIN subtypes, including chandelier cells (CHCs), PV^+^ basket cells (PVBCs), SST;neuronal nitric oxide synthase^+^ cells (SST;NOS1) and SST;CALRETININ^+^ (SST;CR) cells. Importantly, these two TFs are highly expressed in the SST;CR subpopulation (Figures 1E and 1G), suggesting that the broad SST^+^ group of CINs was determined by these TFs. Moreover, it indicated that *c-Maf*, but not *Mafb*, mRNA was detected in VIP^+^ CINs (CGE-derived), suggesting a divergence of *Maf* expression into other CIN progenitor domains.

To complement the single-cell transcriptome analysis data, we performed histochemistry to study *Mafb* and *c-Maf* RNA and protein expression (Figures 1H–1S and S1). Consistent with the scRNA-seq data, we found that MAFB and c-MAF proteins expressed in the MGE SVZ and in MGE-derived immature CINs (SVZ: Figures 1H and 1J, S1A and S1B, S1D and S1E, S1G, and S1J; CINs: Figures 1P–1S, S1C, and S1F). In the MGE SVZ, MAFB and c-MAF were expressed in KI67^+^ SVZ2 progenitors (Figures 1L–1O and S1M–S1AJ’). The SVZ2 is the layer of SVZ progenitors that are between the SVZ1 (adjacent to the VZ) and the layer of newborn neurons. (Petryniak et al., 2007).

CINs tangentially migrate to the cortex along multiple pathways. We explored whether MAFB^+^ and c-MAF^+^ CINs had shared or different trajectories. Immunofluorescence analyses of E15.5 WT neocortices showed that MAFB was expressed in immature CINs migrating along the marginal zone (MZ) pathway and in the cortical plate (CP) (Figures 1P and 1Q), as described in Lim et al. (2018b). On the other hand, c-MAF expression was enriched in the deep migratory pathway (Figures 1R and 1S). This suggests that MAFB and c-MAF may differentially regulate CINs migrating along the superficial and deep pathways, respectively.

### Combined Loss of *Mafb* and *c-Maf* Results in Decreased MGE-Derived CINs

The functions of *Mafb* and *c-Maf* in CIN development remain largely unknown, in part because constitutive *Mafb* and *c-Maf* mutant mice die at embryonic or neonatal ages (Blanchi et al., 2003; Kawauchi et al., 1999). Thus, to examine the prenatal and postnatal functions of these TFs in MGE-derived CINs, we generated conditional mutant mice with floxed *Mafb and c-Maf* (Wende et al., 2012; Yu et al., 2013), combined with *Nkx2.1-Cre* (Xu et al., 2008) alleles, whose expression in the MGE and POA begins on embryonic day (E) ~9.5. We crossed either *Mafb^Flox^* and/or *c-Maf^Flox^* mice to those harboring *Nkx2.1-Cre* and the *Ai14* allele (Madisen et al., 2010), which expresses the fluorescent protein tdTomato after CRE recombination, and subsequently generated both conditional *Mafb* and *c-Maf* single (cKOs) as well as conditional double knockouts (cDKOs). Mice were born at expected Mendelian ratios and lived into adulthood. Of note, these crosses generated both WT and conditional heterozygous states for each genotype (see details in STAR Methods). We did not detect gross phenotype differences between WT and conditional heterozygous mice. Thus, controls used in this report are either WT or mixed conditional heterozygotes, unless otherwise noted.

**Figure 2.**
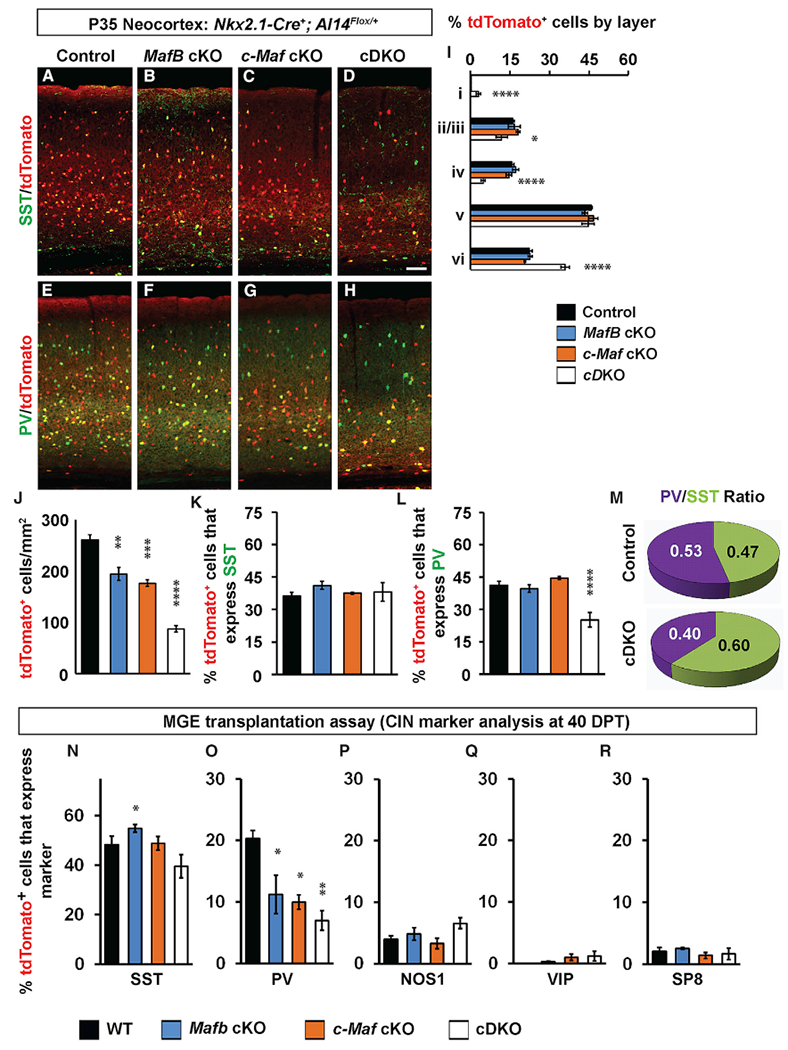
CINs in *Maf* cDKOs Are Reduced in Numbers, Have Altered Laminar Positions, and Have a Decreased Proportion of PV^+^ CINs Cell Autonomously (A–D) Immunofluorescent images from P35 somatosensory cortices from 4 genotypes that show native tdTomato merged with somatostatin (SST) staining. (E–H) Immunofluorescent images from P35 somatosensory cortices from 4 genotypes that show native tdTomato merged with parvalbumin (PV) staining. (I) Quantification of the relative proportion of tdTomato cells that occupy cortical layers. (J) Quantification of the number of tdTomato^+^ cells/mm^2^ in the somatosensory cortex. (K and L) Quantification of the proportion of tdTomato^+^ cells that co-express either SST (K) or PV (L). (M) Pie chart of PV^+^/SST^+^ ratio of the remaining tdTomato^+^ cells. n = 4 for all groups. (N–R) MGE cell transplantation into neocortex assay to assess cell autonomy of CIN phenotypes. Quantification of the number of transplanted tdTomato^+^ cells/mm^2^ that co-express MGE or CGE CIN markers including SST (N), PV (O), NOS1 (P), VIP (Q), and SP8 (R). n = 4 for all groups. Scale bar in (D) represents 100 μm. Data are expressed as the means ± SEM. *p < 0.05, **p < 0.01, ***p < 0.001, ****p < 0.0001.

We began our phenotypic analysis of *Maf* mutant mice by assessing MGE-derived CINs and CIN subgroups from *Nkx2.1-Cre*-lineages (tdTomato^+^) at P35 in the somatosensory cortex (Figure 2). *Mafb* and *c-Maf* cKOs had modest decreases of 25% and 32%, respectively, in tdTomato^+^ cells (Figures 2A–2C, 2E–2G, and 2J; *Mafb* cKOs, p = 0.002; *c-Maf* cKOs, p = 0.0002). On the other hand, *Maf* cDKOs had a 64% reduction in the density of tdTomato^+^ cells (Figures 2D, 2H, and 2J; p < 0.0001). These results suggest that *Mafb* and *c-Maf* have compensatory roles in regulating the number of MGE-derived CINs.

Next, we assessed the proportion of cells that occupied each lamina of the somatosensory cortex. *Mafb* and *c-Maf* cKOs did not differ from controls, whereas the cDKOs had a greater loss in upper lamina (layers II—IV) and an increased proportion in the deep lamina (layer VI) (Figure 2I, layers II/III, p = 0.03; layer IV, p < 0.0001; layer VI, p < 0.0001), even though tdTomato^+^ cell densities were reduced in all layers (Figure 2J). Interestingly, in the cDKOs, MGE-derived CINs were also found in layer I, a lamina that is not populated by those cells (Figure 2I; p < 0.0001).

To determine whether loss of *Mafb* and *c-Maf* affected MGE lineage CINs equally, we determined the proportion of remaining tdTomato^+^ cells in the mutants that expressed either SST or PV. Although the density of tdTomato^+^;SST^+^ CINs decreased in all KOs (Figures 2A–2D; Table S2), the proportion of SST^+^ CINs was similar between genotypes (Figures 2J and 2K). Furthermore, the density of tdTomato^+^;PV^+^ CINs decreased in all KOs (Figures 2E–2H, Table S2); however, the proportion of tdTomato^+^ cells expressing PV was reduced 2-fold only in the cDKO (Figures 2J and 2L; p < 0.0001). This disproportionate decrease in PV^+^ CINs led to an increase in the ratio of SST to PV CINs in the cortex in cDKOs (Figure 2M). Of note, interneurons in the hippocampus and striatum also exhibited similar disproportionate decreases in the PV^+^ cells (Table S2).

In sum, *Mafb* and *c-Maf* together are required for controlling the appropriate number of MGE-derived CINs at P35. Furthermore, they are particularly important in promoting PV^+^ cortical, hippocampal, and striatal interneuron generation and/or maturation.

### Cell Autonomous Role for *Mafb* and *c-Maf* in Establishing Normal Numbers of PV^+^ MGE-Lineage CINs

To test whether the preferential reduction of PV^+^ cells in the *Maf* cDKOs was cell autonomous, we used an MGE transplantation assay to transduce small numbers of MGE progenitor cells into a WT cortex and monitor their development *in vivo* (Vogt et al., 2015). E13.5 *Ai14^Flox/+^* MGE cells, which were either WT, *Mafb^Flox/Flox^, c-Maf^Flox/Flox^*, or *Mafb^Flox/Flox^;c-Maf^Flox/Flox^*, were harvested and virally transduced with a *Cre* expression vector using the *DlxI12b* enhancer. These MGE cells were transplanted into P1 WT neocortices; 40 days later, they were analyzed for SST, PV, NOS1, VIP, and SP8 expression.

The proportion of tdTomato^+^ cells that were PV^+^ were decreased in all *Maf* mutant cells (Figure 2O; *Mafb* cKOs, 45%, p = 0.04; *c-Maf* cKOs, 50%, p = 0.02; cDKOs, 65%, p = 0.006). SST numbers were not changed, except for *Mafb* cKO (increased 14%; Figure 2N; p = 0.04). Other MGE-derived CINs, such as NOS1^+^ cells (Figure 2P), and CGE-derived CINs, VIP^+^, and SP8^+^, were unchanged (Figures 2Q and 2R).

These data suggest that the decrease in PV^+^ MGE-lineage CINs is cell autonomous and that loss of *Maf*s does not lead to CIN fate change from the MGE type (SST and PV) to the CGE type (SP8 and VIP).

### cDKOs Have Reduced CINs and Excess Hippocampal Interneurons at P0

To identify the onset in the reduction of MGE-lineage CINs observed in cDKOs (Figure 2), we assessed tdTomato^+^ cells at multiple ages (Figure S2). At E13.5 and E15.5, we found no significant changes in the density of tdTomato^+^ neocortical cells (Figures S2A–S2H and S2W). By P0, the cDKOs had a decrease (~26%) in the density of tdTomato^+^ neocortical cells (Figures S2I–S2L and S2W; p = 0.046). By P7 (Figures S2M–S2P) and P16 (Figure S2Q–S2T), there were further decreases in the density of tdTomato^+^ neocortical cells, which reached ~64% reduction at P35 (Figure S2W).

Concomitant with the significant decrease in the density of neocortical tdTomato^+^ cells starting at P0, the cDKOs had a ~42% increased density of tdTomato^+^ cells in the hippocampus (Figures S3F–S3J; p = 0.007). However, the increase of hippocampal tdTomato^+^ cells was transient; it was no longer present at P16 (Table S2). By P35, the cDKOs had a comparable reduction of tdTomato^+^ cells in both the hippocampus and somatosensory cortex (Table S2).

We suggest that the transient increased density of tdTomato^+^ cells in the hippocampus could be due to an “over-migration” of CINs into that region, which may contribute to the reduction in their numbers in the neocortex.

### MGE Proliferation and CIN Apoptosis Are Not Altered in cDKOs

Several mechanisms could lead to the reduction of CIN numbers (Figures 2 and S2). Although ectopic cell accumulation in the hippocampus could account for cell loss in the neocortex, it is also possible that reduced cell proliferation and/or increased cell death has a part. We focused our subsequent analyses on the cDKOs. To study MGE proliferation, we administered 5-ethynyl-2′-deoxyuridine (EdU) for 30 min to E13.5 pregnant mice to label the S-phase progenitors (schema, Figure S4A). We assessed EdU^+^ cell density in the VZ, SVZ1, and SVZ2 (secondary progenitors) and found no change in the cDKOs. (Figures S4B–S4D). Next, at E13.5 and E15.5, we determined the density of phospho-histone-3 (PH3^+^) cells to assess the numbers of M-phase progenitors and, again, found no difference (Figures S4E–S4J). These data suggested that, during peak MGE CIN generation, there were no significant differences in the number of S-phase and M-phase progenitors in the VZ and SVZ.

To determine whether increased apoptosis could contribute to the CIN reduction in cDKOs, we stained for cleaved caspase-3 (CC3), a marker of apoptosis. At E13.5 and E15.5, no differences in CC3^+^ cell densities were detected in the MGE or along the CIN migration route. At P0, P7, and P16, we also observed no differences in the density of tdTomato^+^/CC3^+^ cells (data not shown).

Additionally, we stained for IBA1, a marker of active microglia, to see whether increased microglia activity might contribute to the CIN reduction in cDKOs. Again, we observed no significant changes in IBA1^+^ microglia density in the neocortex at P7 and P16 (data not shown). Thus, we did not obtain evidence for increased apoptosis and microglia engulfment contributing to the reduction in neocortical CINs in cDKOs.

### Early Born MGE Lineages Are Overrepresented in cDKO Adult Brains

In the adult (P35) cDKO somatosensory cortex, the remaining CINs maintained the normal proportion of SST^+^ CINs but showed a reduction of PV^+^ CINs. Furthermore, cDKO CINs were disproportionately reduced in superficial layers (likely late born) and increased in deep layers (likely early born) (Figure 2I).

**Figure 3.**
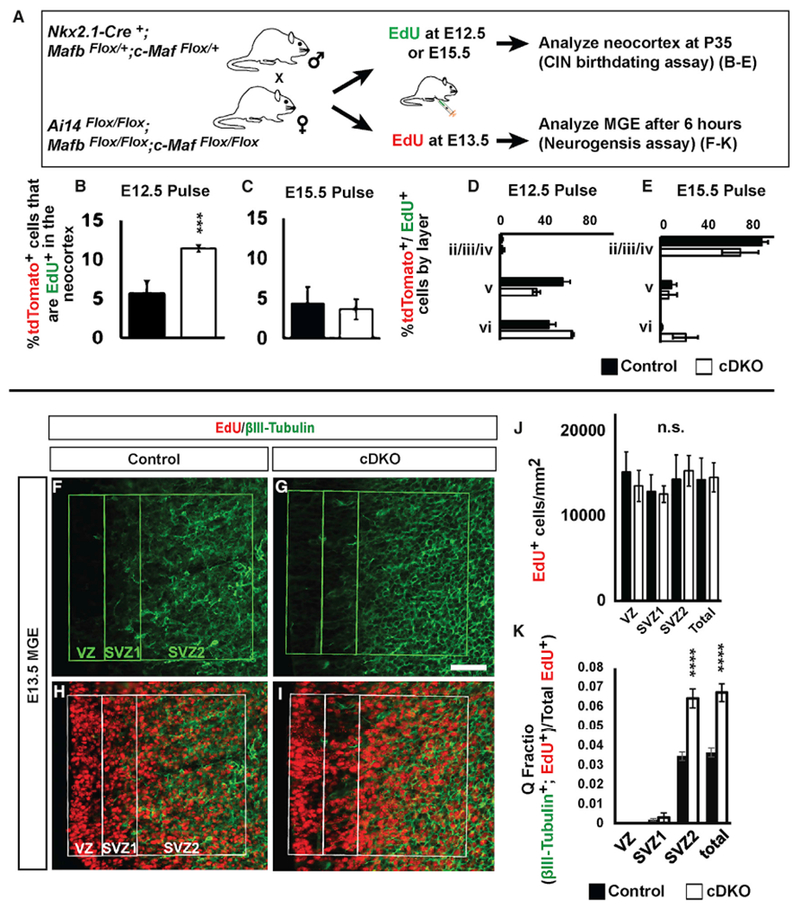
EdU Pulse-Chase Experiments Revealed *Maf* cDKOs Have Increased Early Born Adult CINs and Have Precocious MGE Neurogenesis (A) Schema depicting the EdU pulse-chase assays. (B–K) To follow CIN birthdates (B–E), EdU was injected into pregnant females when embryos were E12.5 or E15.5. The somatosensory cortex was assessed at P35. To study MGE neurogenesis (F–K), EdU was injected into pregnant females when embryos were E13.5; the embryos were analyzed 6 h later for co-expression of EdU and βIII-Tubulin, a neuronal marker. (B and C) Quantification of EdU^+^;tdTomato^+^ double-labeled cells/mm^2^ from EdU pulses at either E12.5 (B) or E15.5 (C). (D and E) Quantification, as a function of cortical lamina, of the proportion of EdU^+^; tdTomato^+^ double-labeled cells from EdU pulses at E12.5 (D) or E15.5 (E). n = 4 for all groups. (F–I) Immunofluorescent images show the MGE co-stained with EdU (H and I) and βIII-tubulin (F and G) from control and cDKO. Boxed region indicates VZ, early SVZ (SVZ1), and late SVZ (SVZ2). (J) Quantification of EdU^+^ progenitors/mm^2^ in the VZ, SVZ1, and SVZ2. (K) Quantification of the Q fraction (EdU^+^; βIII-tubulin^+^/EdU^+^) in the VZ, SVZ1, and SVZ2. n = 3 for all groups. Data are expressed as the means ± SEM. ***p < 0.001, ****p < 0.0001. Scale bar in (G) represents 50 μm. SVZ1, subventricular zone 1; SVZ2, subventricular zone 2; VZ, ventricular zone.

We hypothesized that P35 cDKOs CINs were enriched for early born SST CINs. The birth date of MGE-derived CINs correlates with their cell fate and laminar position. Early born (E12.5–E13.5) MGE cells tend to occupy deep neocortical lamina and express SST, whereas later-born (E15.5) cells tend to occupy superficial lamina and express PV (Inan et al., 2012; Miyoshi et al., 2007; Pla et al., 2006). To test that hypothesis, we followed the fate of early and late-born control and cDKO MGE CINs by giving EdU at either E12.5 or E15.5 and, then, analyzing their neocortical laminar position at P35 (schema, Figure 3A). After the E12.5 EdU pulse, we detected a 2-fold increase in the proportion of double-labeled tdTomato^+^;EdU^+^ cells in cDKOs (Figure 3B; p = 0.0003). However, no differences were observed after the E15.5 pulse (Figure 3C). These data support the idea that the cDKO generates disproportionally more CINs by E12.5. The laminar distribution of tdTomato^+^;EdU^+^ CINs in these experiments showed no major differences, except for a trend for an increase in layer VI and a decrease in layers II/II/IV (Figures 3D and 3E).

Of note, the above data could be confounded by the cell loss that occurs in the cDKO neocortices by P35 (Figure 2). To circumvent that, we used a prenatal, 6 hr, EdU pulse-chase paradigm (schema, Figure 3A) to compare neurogenesis in the control and cDKOs MGE before cell loss occurs. To that end, pregnant mice were given EdU when the embryos were E13.5; 6 hr later the embryos were sacrificed, and immunofluorescently co-labeled for EdU and the neuronal marker, βIII-tubulin (Figures 3F–3I). We quantified the percentage of EdU^+^ βIII-tubulin^+^ double-positive cells to determine the fraction of cells that left the cell cycle and became immature neurons (the quiescent (Q)-fraction) (Figure 3K) (Takahashi et al., 1996). We focused on the MGE SVZ, where newly generated neurons are present before they migrate.

As in previous experiments, we did not detect differences in the density of EdU^+^ cells in the MGE (Figure S4D). However, the proportion of newly post-mitotic (βIII-tubulin^+^;EdU^+^) cells (Q-fraction) generated at E13.5 increased ~2-fold in the cDKOs (Figure 3K; p = 0.0009). These data provided evidence that, although loss of *Mafb;c-Maf* did not change the rate of proliferation, the newly generated cells were becoming neurons at an increased rate in the cDKO. This could lead to an increase in SST^+^ CINs, perhaps at the expense of the PV^+^ CINs.

### Excessive Production of SST^+^ CINs in cDKOs

**Figure 4.**
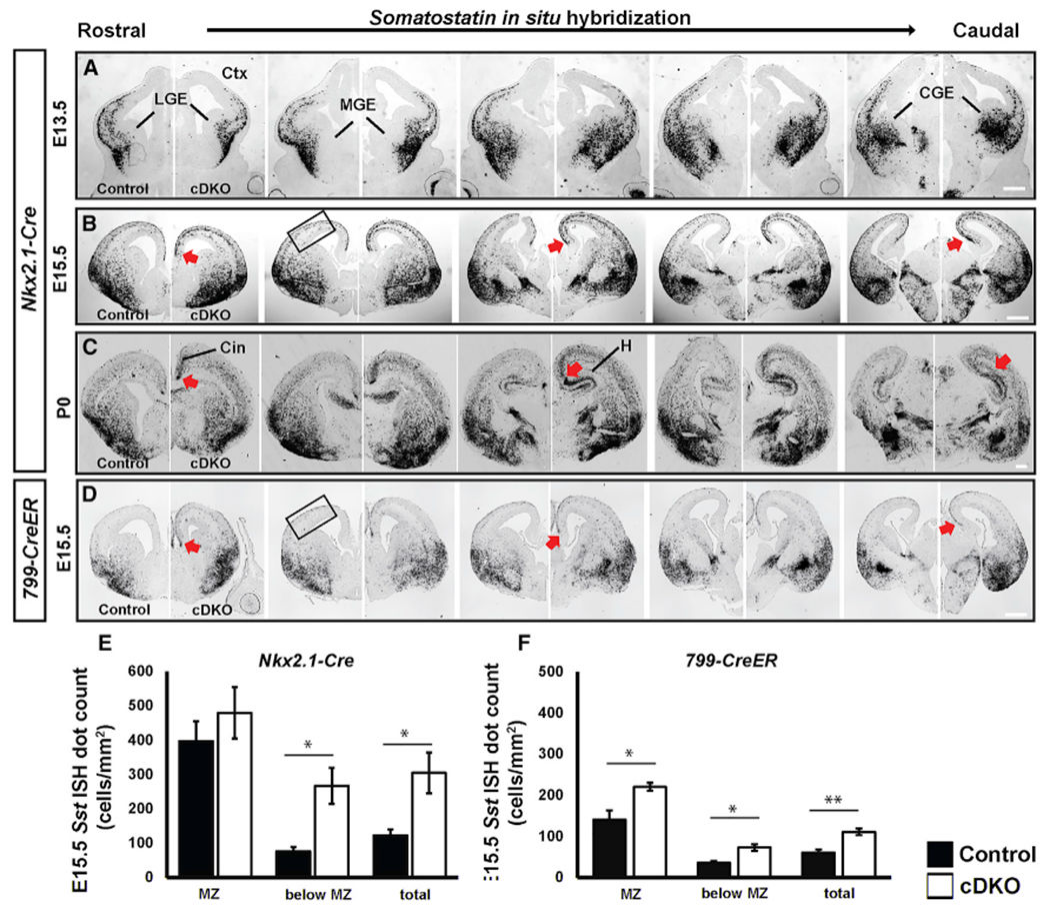
*Mafb* and *c-Maf* Regulate the Quantity and Position (Laminar and Regional) of MGE-Derived *Somatostatin*^+^ Interneurons during Cortical and Hippocampal Development (A–C) *Sst* *in situ* hybridization at E13.5 (A), E15.5 (B), and P0 (C) in control and cDKO generated using *Nkx2.1-Cre.* (D) Sst *in situ* hybridization at E15.5 in control and cDKO generated using *799-CreER*, whose activity begins in the SVZ. Increased numbers of *Sst*^+^ CINs in the dorsomedial cortex and hippocampus are denoted by red arrows. (E) Quantification of *Sst*^+^ CINs/mm^2^ by region in the neocortex in control and cDKO generated with *Nkx2.1-cre.* Increased *Sst*^+^ CINs were observed in laminae below the marginal zone (MZ) in cDKO at E15.5. n = 4 for control; n = 3 for cDKO (F) Quantification of *Sst*^+^ CINs/mm^2^ by region in the neocortex in control and cDKO generated with *799-CreER.* Increased *Sst*^+^ CINs were observed in the MZ and below the MZ in cDKO at E15.5. n = 4 for both groups. Data are expressed as the means ± SEM. *p < 0.05, **p < 0.01. Scale bar in (A)–(D) represents 500 μm. Boxed region indicates where quantification was done. Cin, cingulate cortex; MGE, medial ganglionic eminence; LGE, lateral ganglionic eminence; H, hippocampus

Our earlier EdU pulse-chase experiments provided evidence that cDKOs have an increased proportion of early born CINs in the adult cortex (Figure 3B), as well as increased MGE neurogenesis at E13.5 (Figure 3K). Thus, because the early wave of CINs are mostly *Sst*^+^ (Inan et al., 2012; Miyoshi et al., 2007; Pla et al., 2006), we were interested in determining whether there were increased numbers of *Sst*^+^ cells being generated in the cDKO MGE. We characterized *Sst* RNA expression by *in situ* hybridization (ISH) at E13.5, E15.5, and P0 (Figures 4A–4C). Although there was no detectable change in *Sst* expression at E13.5 (Figure 4A), there was an ~2-fold increase in the density of cortical *Sst*^+^cells at E15.5 (Figures 4B and 4E; p = 0.0184). This increase was even more apparent by P0 (Figure 4C). Furthermore, at P0, *Sst*^+^ cells were clearly increased in the hippocampus (caudal) and in the cingulate (rostral) cortex (Figure 4C), consistent with the transient increase in neonatal MGE-lineage cells (tdTomato^+^) in those locations at P0 (Figures S3F–S3J).

**Figure 5.**
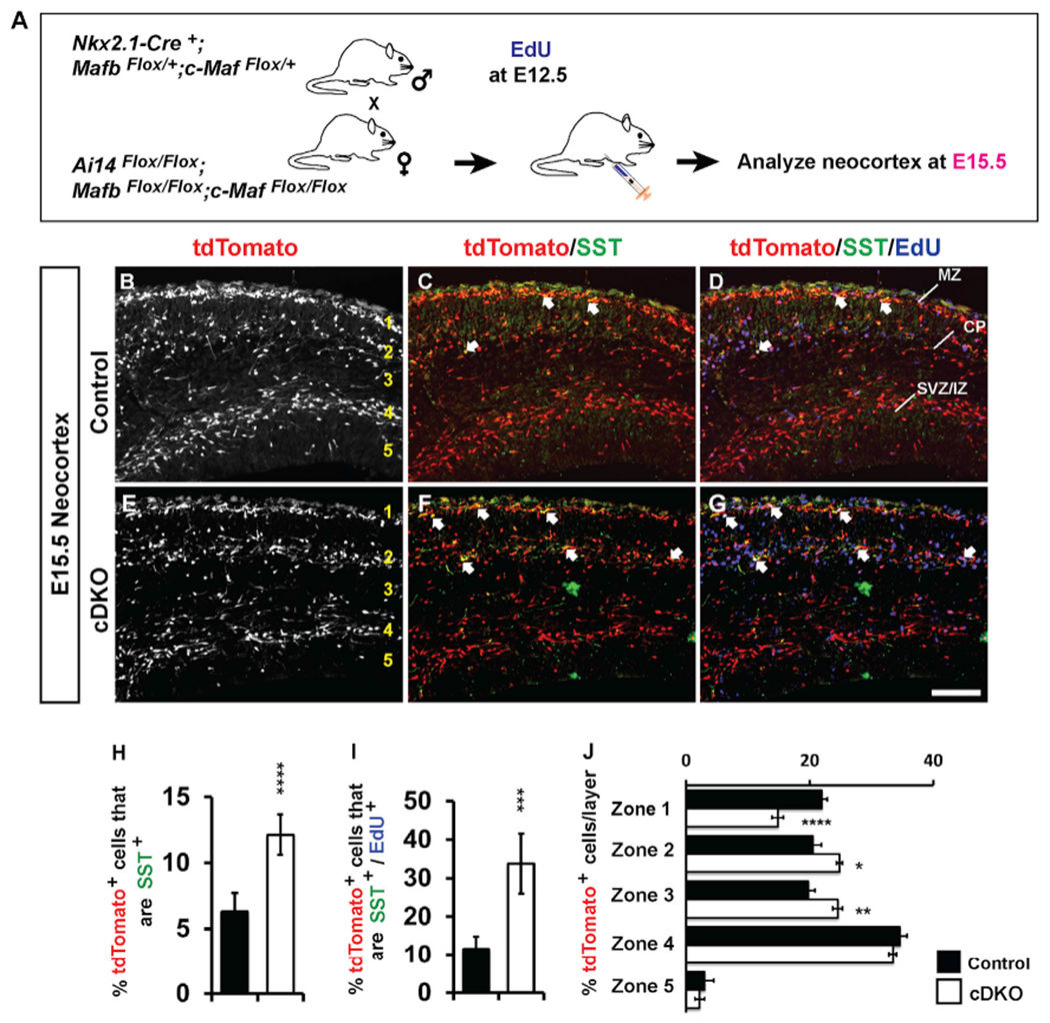
Increased Numbers of SST^+^ Interneurons in Developing cDKO Neocortex (A) Schema depicting the EdU 3-day pulse-chase experiment. EdU was injected into pregnant females when embryos were E12.5. The neocortices were assessed at E15.5. (B–G) Immunofluorescent images of E15.5 neocortices showing tdTomato^+^ (B and E) cells co-stained for SST (C and F) and EdU (D and G). Arrows show double- or triple-labeled cells (C, D, F, and G). (H) Quantification of the number of tdTomato^+^ cells/mm^2^ that are SST^+^. (I) Quantification of the number of EdU-labeled tdTomato^+^ cells/mm^2^ that are SST^+^. (J) Quantification of the proportion of tdTomato^+^ cells for the five layers labeled in (B) and (E). Marginal zone (zone 1), cortical plate and subplate (zone 2), intermediate zone (zone 3), deep migration stream (zone 4), and the ventricular zone (zone 5). n = 3–4 for all groups. Data are expressed as the means ± SEM. *p < 0.05, **p < 0.01, ***p < 0.001, ****p < 0.0001. Scale bars in (G) represents 100 μm.

To determine whether the increase in *Sst*^+^ cells in the cortex was related to the precocious neurogenesis observed at early ages in the MGE (Figure 3K), we administered EdU to pregnant mice at E12.5 and analyzed them at E15.5 in the neocortex to assess the number of SST^+^ and tdTomato^+^ cells generated at an earlier age (Schema, Figures 5A–5G). Indeed, the proportion of tdTomato^+^ cells that expressed SST nearly doubled in cDKOs (Figure 5H; p < 0.0001). Moreover, we found that the number of triple-labeled tdTomato^+^;SST^+^ CINs that were EdU^+^ also doubled in the cDKOs (Figure 5I; p = 0.0002). Overall, these data show that without *Mafb* and *c-Maf*, the MGE has a normal density of progenitors, which generate more early born SST^+^ CINs. Because there was no increased density in cortical tdTomato^+^ cells at E15.5 (Figure S2), we hypothesize that the remaining tdTomato^+^;SST^−^ cells are most likely of the PV^+^ lineage (although that age is too young to assess PV expression).

### *Mafb* and *c-Maf* Function in SVZ Progenitors to Control MGE-Derived CIN Production

We have provided evidence that the lack of *Mafb* and *c-Maf* results in Sst^+^ CIN overproduction in the late SVZ of MGE (SVZ2; Figure 3K). Thus, we hypothesized that *Mafb* and *c-Maf* function in the SVZ to control the production and fate of CINs. To further test that idea, we generated conditional *Maf* cDKOs using the *799-CreER* mouse line, whose activity initiates in the SVZ of the MGE (Silberberg et al., 2016).

First, we identified, in more detail, the type(s) of MGE progenitors in which *799-CreER* activity is initiated (Figure S3). We induced *Cre* activity with a tamoxifen injection at E11.5 and assayed CRE activity with the *Ai14* reporter. We harvested the embryos at E12.5, 30 min after an EdU injection. To test whether there was *799-CreER* activity in the MGE progenitors, histological sections were analyzed with antibodies to tdTomato, EdU (S-phase progenitors) and Ki67 (pan-progenitor marker). We identified tdTomato and Ki67 double-positive cells in the SVZ2 but not in the VZ or SVZ1 (Figures S3K and S3L). Thus, we provided evidence that *799-CreER* activity initiates in a subset of MGE “late” progenitors. Furthermore, MAFB and tdTomato co-localize in the MGE SVZ2, suggesting MAFB expression initiates around the time that *799-CreER* activity begins (Figures S3M–S3O).

Next, we used *799-CreER* (tamoxifen at E11.5) to generate cDKO embryos, which were analyzed at E15.5 to test whether the mutation affected the number of SST^+^ CINs. Sst ISH showed an ~2-fold increase (Figures 4D and 4F; p = 0.003), which was also observed with *Nkx2.1-Cre*-mediated deletion (Figures 4B and 4E); similar changes were also seen in CIN laminar distribution (Figures 4E and 4F; MZ, p = 0.03; below MZ, p = 0.012).

Lastly, we used *Sst-IRES-Cre* (Taniguchi et al., 2011) and Ai14 (Figures S3P–S3Y) to generate cDKO mice that express tdTomato at P0 and P30 to test whether deletion of *Mafb* and *c-Maf* expression in post-mitotic SST^+^ lineage cells affects the numbers of SST^+^ CINs. In the cDKOs, we observed no change in the density of tdTomato^+^ CINs or tdTomato^+^;SST^+^ CINs (data not shown).

Thus, the use of *799-CreER* and *Sst-IRES-Cre* support the conclusion that MAF proteins act in the SVZ2, and not in immature CINs, to repress the generation of SST^+^ CINs.

### *c-Maf* Controls CIN Migration Alone and Together with *Mafb*

*Mafb* and *c-Maf* cDKOs, in addition to the increase in *Sst*^+^ CINs in immature neocortex and hippocampus, also have changes in CIN migration patterns at E15.5. We quantified CIN densities of the deep and superficial migration streams using both E15.5 Sst ISH (Figures 4E and 4F) and E15.5 tdTomato immunofluorescence (IF) (Figure 5J).

In the *Nkx2.1-Cre*-mediated cDKO, the density of *Sst*^+^ cells in the lamina below the MZ was increased (Figure 4E; p = 0.02). Consistent with that, the proportion of tdTomato^+^ CINs in the MZ (zone 1) was reduced ~30% (Figure 5J; p < 0.0001). In turn, zones 2 and 3, which included the CP and subplate, had increased proportions of cells in the cDKOs (Figure 5J; p = 0.04 and 0.003, respectively), whereas the deeper layers—zones 4 and 5—were unchanged.

We also conducted Sst ISH at E15.5 in the *Mafb* and *c-Maf* single cKOs (Figures S3A–S3D). Although there was no obvious change in total *Sst*^+^ cell density in the *Mafb* cKO, the *c-Maf* cKO showed a slight increase (Figure S3E).

Of note, tdTomato expression identified an alteration in the laminar pattern of CINs in the deep tangential migration zone at E15.5 in the *c-Maf* cKO and the cDKO. Although control and *Mafb* cKOs have tightly organized deep migration zones (arrow-heads, Figures S2E and S2F), that region is disorganized and has ~2-fold fewer CINs in the *c-Maf* cKOs and the cDKOs (Figures S2G and S2H; *c-Maf* cKO, p < 0.0001; cDKO, p = 0.0001). The observation that the *c-Maf* cKO has a defect in the deep migration layer correlates with the *c-Maf* selective expression in that location (Figures 1P and 1R). Together those alterations in CIN laminar organization, during their tangential migration, suggest that *c-Maf* alone, and together with *Mafb*, control migration of immature CINs.

To understand the mechanisms underlying the migration phenotypes, we assayed *Cxcr7* RNA expression because *Cxcr7* mutants have a reduction in migrating CINs in the MZ and an increase in the CP, very similar to the phenotype observed in the distribution of tdTomato^+^ CINs in the cDKO (Figure 5J). Indeed, in the cDKO, the cortical distribution of *Cxcr7* RNA resembled that of tdTomato (Figures S5O, S5P, S5PO’, and S5P’) (Li et al., 2008; Sánchez-Alcañiz et al., 2011; Stumm et al., 2003; Wang et al., 2011). This provides evidence that *Mafb* and *c-Maf* control CIN migration independent of *Cxcr7* expression, potentially through a parallel pathway and/or downstream event.

### *Mafb* and *c-Maf* Loss of Function Alters CIN Action Potential Firing Properties and Synaptic Excitation

To better understand how*Maf* mutations affect CINs physiology, we performed whole-cell patch-clamp recordings from *Nkx2.1-Cre*-lineage CINs in cortical layer 5 of the somatosensory cortex in adult control and *Maf* mutant mice (P63–P82). CINs were visually identified for electrophysiological analyses via tdTomato expression. For assessments, where feasible, we *post hoc* identified fast-spiking (FS) and regular-spiking (RS) CINs, based on their firing patterns, and analyzed their cellular electrical properties separately.

### Active and Passive Membrane Properties of CINs

**Figure 6.**
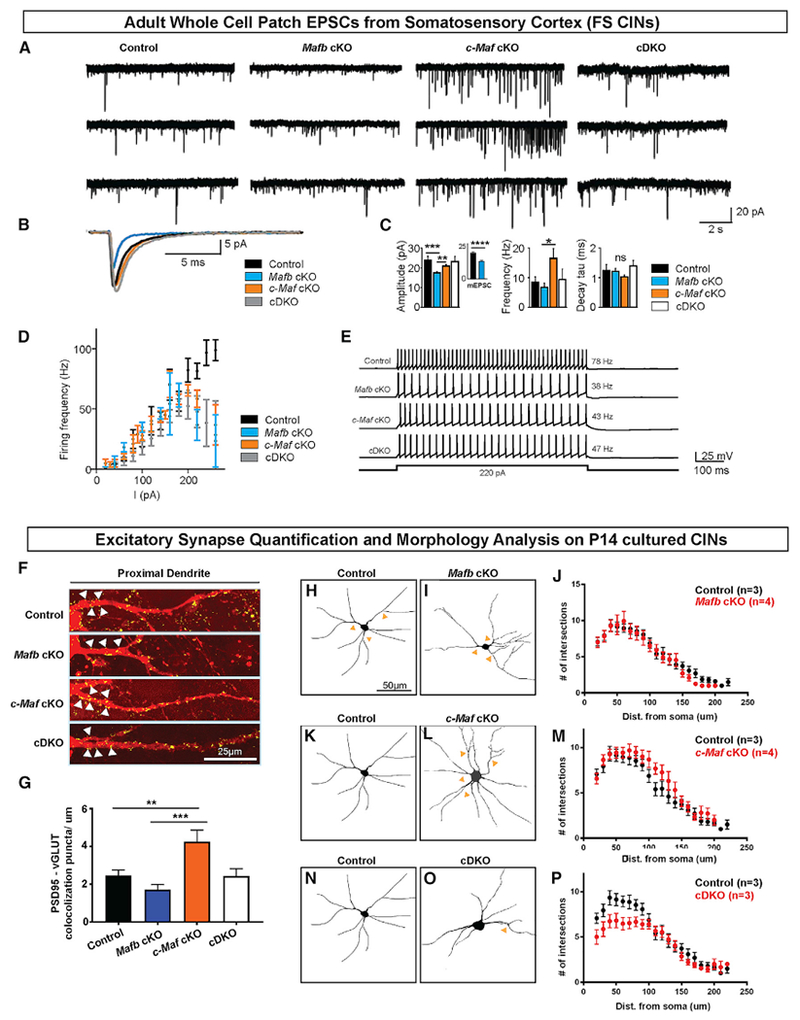
Synaptic Excitation and Intrinsic Excitability of Fast-Spiking CINs in *Mafb* cKO and *c-Maf* cKO and cDKO Mice (A) Representative traces of spontaneous EPSCs (sEPSCs) in layer 5–6 fast-spiking (FS) CINs. (B) Overlaid average sEPSCs from the representative cells depicted in (A). Note the reduced amplitude of sEPSC specifically in *Mafb* cKO FS CINs. (C) Quantification (means ± SEM) of the amplitude, frequency, and decay time constant of sEPSCs (V_hold_ = −70 mV) in FS CINs. Note the reduced amplitude of sEPSCs in *Mafb* cKO compared with other genotypes and the enhanced frequency of sEPSCs in *c-Maf* cKO compared with other genotypes. *p < 0.05; **p < 0.01 (control, 28 cells; *Mafb* cKO, 36 cells; *c-Maf* cKO, 39 cells; cDKO, 12 cells). Inset indicates reduced amplitude of mEPSCs in *Mafb* cKO compared with control (control, 24 cells; *Mafb* cKO, 16 cells; ***p < 0.001, Mann-Whitney test). (D) F–I curve for FS CINs—a plot of the mean action potential firing frequency as a function of current intensity injected in the FS CINs (control, 19 cells; *Mafb* cKO, 32 cells; *c-Maf* cKO, 43 cells; cDKO, 12 cells). Note the inability of all *Maf* mutant FS CINs to sustain firing frequencies greater than ~60 Hz, compared with control CINs that can exhibit firing rates >100 Hz. p < 0.0001 for all genotypes. (E) Representative firing traces from FS CINs for each genotype. (F) Confocal immunofluorescence analysis of tdTomato^+^ CINs grown *in vitro* for 14 days (14 DIV). Representative flattened Z-plane images of excitatory synapse labeling in proximal dendrites of tdTomato^+^ CINs from 4 genotypes. Yellow dots represent the colocalized punta of tdTomato, PSD95, and vGLUT1 staining. (G) Quantification (means ± SEM) of excitatory synapses on proximal dendrites (within 15 μm from soma); n = 3–4 (animals) per genotype and 20–30 proximal dendrites were analyzed per group. **p < 0.01; ***p < 0.001. (H and I) Representative images of control (H) and *Mafb* cKO (I) CINs at DIV14. (J) Quantification (means ± SEM) of Sholl analysis between control and *Mafb* cKO CINs. (K and L) Representative images of control (K) and *c-Maf* cKO (L) CINs at DIV14. (M) Quantification (means ± SEM) of Sholl analysis between control and *c-Maf* cKO CINs. (N and O) Representative images of control (N) and cDKO (O) CINs at DIV14. (P) Quantification (means ± SEM) of Sholl analysis between control and cDKO CINs. Note the increase in neurite complexity in *c-Maf* cKO and decrease in neurite complexity in cDKO. n = 3–4 (animals) for all groups; each genotype had 15–20 cells analyzed. Scale bar in (F) represents 25 μm and in (H) represents 50 μm.

We measured the mean firing frequency of FS and RS CINs in response to the increasing intensity of the intracellular positive current injection (F–I plots, Figure 6D). We found that, in FS cells, the maximal firing frequency was reduced in all mutant CINs compared with controls. FS CINs from all *Maf* mutants could not sustain firing rates greater than ~60 Hz and could not maintain firing rates for currents above 200 pA, most prominently in the *Mafb* cKOs. Interestingly, the F–I plots in RS CINs were similar between controls and all *Maf* mutants (Figure S6B). These results provide evidence that loss of *Mafb* and *c-Maf* reduces the ability of FS CINs to sustain action potential (AP) firing.

In the FS CIN population, we found similar passive electrical membrane properties when comparing controls with each of the *Maf* mutants (Table S3, top). We also observed no strong changes in single AP properties (threshold, amplitude, and duration) in *Maf* mutants (Table S3, bottom). However, there were differences when *Mafb* and *c-Maf* cKOs were compared side-by-side (Table S3, bottom). Notably, *Mafb* cKO had lower AP threshold (p = 0.001 versus *c-Maf* cKO); increased AP amplitude (p = 0.025 versus *c-Maf* cKO); increased AP duration (p = 0.04 versus *c-Maf* cKO); and increased AP half duration (p = 0.02 versus *c-Maf* cKOs). These results suggest *Mafb* and *c-Maf* have divergent roles in regulating CIN AP properties.

In the RS CIN population, some passive electric membrane properties were different in *Maf* mutants (Table S3, top). For instance, *Mafb* cKOs had a more depolarized resting membrane potential (p = 0.01 versus control); *c-Maf* cKOs had decreased membrane capacitance (p = 0.04 versus control); and cDKOs had decreased input resistance (p = 0.0005 versus control). We observed similar AP properties between controls and all *Maf* mutants (Table S3, bottom). These results suggest that the features of RS CINs are also altered after deletion of *Mafb* and *c-Maf*, but the changes may not be as extensive as the changes seen in FS CINs.

### Excitatory Synaptic Properties onto CINs

To investigate excitatory inputs onto CINs, we measured the spontaneous excitatory post-synaptic currents (sEPSCs) onto control and *Maf* mutant CINs (Figure 6). sEPSCs were smaller in amplitude in *Mafb* cKO compared with control *c-Maf* cKOs and cDKOs (Figures 6A and 6C; Table S4; p = 0.0004, 0.002, and 0.002, respectively), whereas the average frequency of sEPSCs was increased in *c-Maf* cKO compared with *Mafb* cKO (Figures 6A and 6C; Table S4; p = 0.015). These phenotypes were primarily observed in FS CINs. We found no significant differences in the decay time constant of the recorded sEPSCs across different genotypes (Table S4). These results suggest that the synaptic excitatory input is reduced onto *Mafb* cKO CINs but increased onto *c-Maf* cKO CINs.

**Figure 7.**
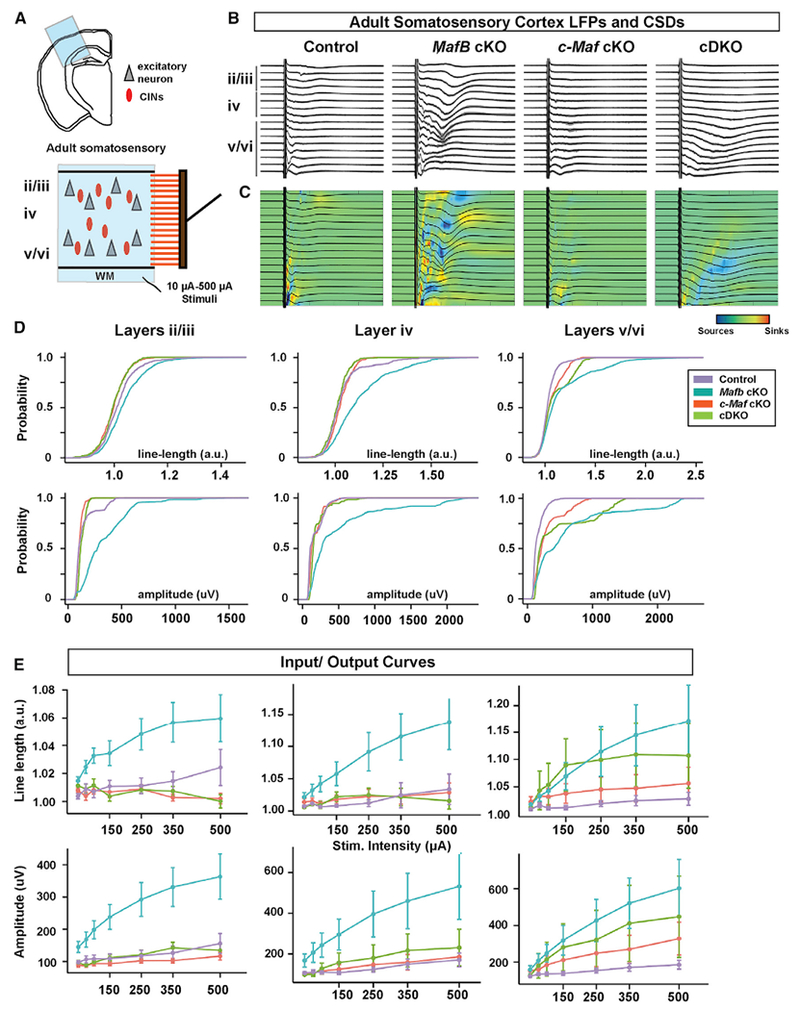
LFPs and CSDs for Neocortical Slices Illustrate Cortical Hyper-Excitability in *Mafb* cKOs but Not in *c-Maf* cKOs or DKO Mice (A) Schema depicting the local field potential (LFP) multi-array experiment. (B) Examples of average LFPs (black) overlaid on the individual LFPs recordings (gray) for representative slices. (C) Current source densities (CSDs) for the average LFPs of the representative slices. Blue indicates a source, and red indicates a sink. (D) Bootstrapped cumulative probability distributions of the amplitude and the line-length (see STAR Methods for details) of the LFP evoked by 500 μA stimulation of the white matter. Note that *Mafb* cKOs show greater excitability in all layers at 500 μA (indicated by greater line-length and amplitude in all layers compared with all genotypes), whereas *c-Maf* cKOs show hyporesponsiveness in superficial layers (indicated by reduced line-length and amplitude in layers 2–3 compared with control). (E) Average LFP responses for increasing stimulation intensities of the white matter. Note that average responses for *Mafb* cKOs in all layers show greater intensity-dependent-responses than the other genotypes. Note that only in layers 5–6 DKOs show a trend toward enhanced excitability approaching that of *Mafb* cKOs. 6 controls, 7 *Mafb* cKOs, 6 *c-Maf* cKOs, and 3 cDKOs were used for the analyses. Graphs in (E) are shown as means ± SEM; p values are in Table S5.

To determine whether the reduced sEPSCs amplitude observed in *Mafb* cKO resulted from changes in pre- or post-synaptic mechanisms, we measured miniature EPSCs (mEPSCs) in controls and the *Mafb* cKO CINs. Similar to sEPSCs, we observed no change in the average mEPSC frequency but a significant decrease in mEPSC amplitude in *Mafb* cKO compared with controls (Figure 6C, inset; Table S4, bottom; p < 0.0001). These results suggest that the reduced sEPSC amplitude in the *Mafb* cKO CINs was likely due to a post-synaptic mechanism, rather than a reduced pre-synaptic transmitter release, and was not confounded by the increased cortical activity (Figure 7).

### *c-Maf* cKO CINs Had an Increased Density of Excitatory Synapses and Increased Neurite Complexity *In Vitro*

To determine whether the changes in the *Maf* mutant sEPSCs were due to a change in glutamatergic synapse densities and/or CIN morphology, we grew primary cortical neurons for 14 days from control, *Mafb* cKO, *c-Maf* cKO, and cDKO P0 mice. We then analyzed soma size, neurite complexity (Sholl analysis), and the excitatory synapse density on proximal dendrites of tdTomato^+^ CINs (Figures 6, schema, and S6D). Soma size was similar between controls and all *Maf* mutants (Figure S6E). Sholl analysis revealed that the cDKOs had decreased neurite complexity (Figures 6N–6P), whereas the *c-Maf* cKOs had increased neurite complexity (Figures 6K–6M). Next, we studied excitatory synapses using vGLUT1 and PSD95 as pre- and post-synaptic markers (Figure 6F). CINs from *c-Maf* cKOs had an increased density of excitatory synapses compared with both controls and *Mafb* cKOs (Figure 6G; p = 0.008 and 0.0001, respectively), in agreement with an increased frequency of sEPSCs in *c-Maf* cKO (Figures 6A and 6C). On the other hand, the *Mafb* cKOs showed a trend for reduced density of excitatory synapses compared with controls and a significant decrease compared with *c-Maf* cKOs (Figures 6F and 6G; p = 0.0001). Of note, excitatory synapse density in the cDKOs resembled that of the control, supporting the hypothesis that *Mafb* and *c-Maf* have opposite effects on synaptogenesis, which could have important impacts on physiological phenotypes in the single and double mutants. Notably, the normal excitatory synapse density result in the cDKO was consistent with the finding that sEPSCs were similar between the control and the cDKO (Figures 6A, 6C, and 6G).

### Slice Local Field Potential Analyses Showed *Mafb cKOs* Had Increased Neocortical Circuit Excitability, Unlike the *c-Maf* cKOs and cDKOs

To further assess the effect of the *Maf* mutations on cortical circuit excitability, we measured the local field potentials (LFPs) in acute brain slice preparations from the adult somatosensory cortex. We recorded LFPs across all cortical lamina evoked by electrical stimulation of the white matter tract (schema, Figure 7A). We performed current-source density (CSD) analysis (Aizenman et al., 1996) to determine the patterns of cortical activation between all genotypes. Notably, the CSD pattern was most significantly affected in the *Mafb* cKO neocortex, which showed a pattern consistent with hyper-excitability with an altered spatiotemporal pattern of synaptic sources and sinks, where darker blue and red represent more deviation from normal activation (Figure 7C). In contrast, the CSD pattern of *c-Maf* cKOs suggested that cortical activity was diminished (Figures 7B and 7C). Interestingly, cortical excitability of the cDKOs fell in between that of *Mafb* and *c-Maf* cKOs (Figures 7B and 7C). Quantification of the duration of the response to the stimulus, line-length, and the amplitude of the evoked LFPs across layers show the above differences between *Maf* mutants (Figures 7D and 7E), and detailed statistical comparisons between groups can be found in Table S5.

Thus, LFP analysis provides evidence that the *Mafb* cKO results in cortical circuit hyper-excitability, whereas the *c-Maf* cKO results in cortical circuit hypo-excitability. Notably, the finding that cDKOs have relatively normal excitability in this assay, provides further evidence that *Mafb* and *c-Maf* control excitability in divergent ways.

## DISCUSSION

### *Mafb* and *c-Maf* Control MGE CIN Numbers

*Mafb* and *c-Maf* are expressed in the MGE SVZ and persist in migrating immature and mature CINs, but not in pallidal projection neurons, unlike other known MGE TFs (Hu et al., 2017). Thus, we wondered whether loss of *Mafb* and *c-Maf* together might abort the specification of MGE-derived CINs; however, this was not the case, as cDKOs still generated CINs that tangentially and radially migrated to the neocortex (Figures 2 and S2). This raised the possibility that other TFs are responsible for generating CINs versus projection neurons. These TFs may coordinate with *Lhx6, Nkx2-1, Dlx1/2*, and *Zfhx1b* that have already been shown to contribute to initiating CIN specification (Anderson et al., 1997; McKinsey et al., 2013; Sussel et al., 1999; Zhao et al., 2008).

*Maf* cDKOs have decreased SST^+^ and PV^+^ CINs at P35 with a preferential loss of PV^+^ CINs (Figure 2). In addition, about 40% of the tdTomato^+^ cells in the cDKO do not express SST and PV (in the control group, about 20% of the tdTomato^+^ cells do not express SST and PV, which could be due to antibody labeling efficiency). This could be due to a change in cell fate. We explored whether the ~20% “missing” cells in the cDKO that had an alternative fate. We investigated whether the PV^+^ cells were converted into the *Cck+* basket cells, CGE-type CINs (SP8^+^), other types of INs (NOS1^+^, NPAS1^+^), cholinergic striatal interneurons (ChAT^+^), or oligodendrocytes (OLIG2^+^), but we did not find an increase in these cell types in the cortex of cDKOs (data not shown). Thus, we hypothesize that ~20% of the MGE lineage CINs in the cDKO may be either some other cell type or poorly differentiated PV^+^ CINs. Furthermore, our MGE transplant data support that conclusion (Figure 2). Alternatively, CINs in the cDKO may have impaired maturation or abnormal responses to environmental perturbations, which, in turn, could affect SST and PV expression.

### *Mafb* and *c-Maf* Provide a Brake on Neural Differentiation

*Mafb* and *c-Maf* control proliferation in hematopoietic stem cell, macrophages, and epidermal cells (Lopez-Pajares et al., 2015; Sarrazin et al., 2009; Soucie et al., 2016). Their expression in the SVZ suggests that they could regulate proliferation of secondary progenitors in the MGE. However, we did not observe such changes in the cDKO MGE (Figure S4). On the other hand, in the SVZ of cDKOs, we observed increased expression of βIII-tubulin, a marker of immature neurons at E13.5 (Figures 3I and 3K). This provides evidence that *Mafb* and *c-Maf* restrain neurogenesis. Thus, *Mafb* and *c-Maf*, by serving as a brake on neural differentiation, may regulate cell fate specification.

### *Mafb* and *c-Maf* Repress SST CIN Fate

SST CINs are largely generated before PV CINs (Inan et al., 2012; Miyoshi et al., 2007; Pla et al., 2006). Here, we show that *Mafb* and *c-Maf* control that temporal sequence by restraining the production of SST CINs. The cDKOs generate excessive SST^+^ MGE-derived cells, many of which become CINs. As early as E15.5, there is an obvious increase in immature SST^+^ CINs without any increase in total numbers of MGE-derived cells (Figures 5 and S2). Moreover, we show that, at E15.5, there were increased SST^+^ CINs that were born around E12.5 (Figure 5). Analyses (ISH) of multiple regulators of MGE development did not yield insights into how *Mafb* and *c-Maf* repress SST fate or promote PV fate (Figure S5). Thus, we hypothesize that *Mafb* and *c-Maf* control the timing of when other factors specify SST and PV CIN fate.

### *Mafb* and *c-Maf* Functions Begin in SVZ2 of the MGE to Control CIN Fate

Are SST and PV CINs produced by different MGE progenitors? There is a proposal that SST^+^ CINs primarily arise by direct neurogenesis from radial glial progenitors in the VZ, whereas PV^+^ CINs are produced by secondary progenitors in the SVZ (Petros et al., 2015). Our results are not fully consistent with that model. Given that *Mafb* and *c-Maf* repress the generation of SST CINs, it is likely that these TFs are functioning autonomously in cells that produce SST CINs. *Mafb* and *c-Maf* are expressed in the SVZ and are not detected in the VZ at E12.5 and E15.5 (Figures 1 and S1), arguing that these TFs are repressing SST CIN production in SVZ progenitors.

To further address *Mafb* and *c-Maf* functions in the SVZ, we used the recently generated *799-CreER* line (Silberberg et al., 2016). Here, we demonstrated that *799-CreER* activity begins in the SVZ2 of MGE (Figures S3K and S3L). SVZ2 is thought to contain the most mature progenitors of the ganglionic eminences (Petryniak et al., 2007). We used *799-CreER* to generate *Maf* cDKOs and found that they phenocopy the increase in *Sst*^+^ CINs at E15.5 in the *Nkx2.1-Cre* cDKO (Figure 4). Notably, we did not observe a decrease in tdTomato^+^ CIN numbers using *SST-IRES-Cre*, unlike the *Nkx2.1-Cre*-generated *Maf* cDKOs (data not shown). This provides evidence that *Mafb* and *c-Maf*, in the SVZ2, and not in postmitotic neurons, control the decision between SST and PV CIN fate.

We hypothesize that the MGE SVZ produces both SST and PV CINs and that *Mafb* and *c-Maf* control the probability and timing of SST and PV CIN generation by repressing SST CIN fate and promoting PV CIN fate (model top, Figure S7). Moreover, contrary to our hypothesis, Mi et al. (2018) proposed that *c-Maf* promotes the generation of SST^+^ CINs based on loss-of-function and viral gain-of-function studies. We agree that adult *c-Maf* mutants have reduced SST^+^ CINs (Figure 2; Table S2). However, at E15.5, we did not detect a reduction in *Sst*^+^ CINs in the *c-Maf* cKO (Figure S3E), supporting the idea that the reduction is caused by postnatal CIN loss (Figure S2).

### Interneurons in *Maf* Mutants Show Laminar and Regional Mislocalization

The combined loss of *Mafb* and *c-Maf* partially phenocopies the prenatal lamination defect of *Cxcr4* and *Cxcr7* mutants, which have a depletion of tangentially migrating cells in the MZ and their premature entry into the CP (Figure 5) (Li et al., 2008; Sánchez-Alcañiz et al., 2011; Wang et al., 2011). Thus, we investigated *Cxcr7* expression in the cDKO but found that its expression levels appeared normal at E14.5 (Figure S5).

Lamination defects were also observed in the P35 cDKO cortices (Figure 2); they had greater numbers of CINs in deep (layer VI), and fewer CINs in superficial (layers II–IV) lamina, a phenotype also observed in the *Cxcr7* cKO adult cortex (Wang et al., 2011). This further raises the possibility that the CXCR signaling pathway is regulated by *Mafb* and *c-Maf*.

The cDKOs exhibited an additional migration defect: excessive numbers of MGE-derived INs in the P0 hippocampus (Figures 4 and S3). We suggest that this may reflect a defect in the ability of migratory CINs to detect signals to stop in the neocortex, thus, resulting in continued migration into the hippocampus. However, the *Maf* cDKO interneuron accumulation in the hippocampus diminished over time, and by P35, the hippocampus had reduced tdTomato^+^ cells (Table S2). Thus, both CINs and hippocampal interneurons are reduced over time in *Maf* mutants.

### *Mafb* and *c-Maf* Have Distinct Roles in Regulating Fast-Spiking CINs and Neocortical Circuit Function Postnatally

Our results suggest that *Mafb* and *c-Maf* have distinct functions postnatally in regulating the physiological properties of FS CINs. The finding that sEPSC and mEPSC amplitudes were smaller in the *Mafb* cKO suggests that *Mafb* promotes excitation of FS CINs (Figure 6). This result is consistent with evidence that *Mafb* mutant spiral ganglion neurons had reduced postsynaptic AMPA receptors (Lu et al., 2011; Yu et al., 2013). The reduced excitation of CINs in the *Mafb* cKO could account for the enhanced circuit excitability observed in their LFP recordings (Figure 7). On the other hand, the *c-Maf* cKO CINs had an increased density of excitatory synapses and their sEPSC frequency was enhanced compared with the *Mafb* cKO CINs, suggesting that *c-Maf* represses CIN excitation (Figure 6). The enhanced excitability of CINs in the *c-Maf* cKO could explain the lower circuit excitability in their LFP recordings (Figure 7).

Of note, the cDKO has intermediate phenotypes in the LFP, sEPSC and excitatory synapse analyses. This raises the possibility that *Mafb* and *c-Maf* have distinct transcriptional effects, potentially opposing roles, in maturing and/or mature CINs and cortical circuit excitability (model bottom, Figure S7).

The finding that *Mafb* and *c-Maf* mutations mainly affect FS CINs suggests that these *Maf* genes regulate network excitability mainly by regulating the FS CINs. The fact that *Mafb* and *c-Maf* cKOs as well as cDKOs all have reduced firing of FS CINs in response to current injections (Figures 6D and 6E), but *Mafb* and *c-Maf* mutations have distinct effects on synaptic excitation of these cells (Figure 6C), suggests that the opposing roles of *Maf* genes on cortical network excitability mainly result from their distinct effects on the synaptic, rather than intrinsic, electric properties of CINs. Future experiments, such as electrocorticographic recordings, would be useful in determining how the *Mafb* and *c-Maf* mutations affect neocortical activity and pathophysiology *in vivo.*

In summary, we propose that, in the MGE SVZ, *Mafb* and *c-Maf* have redundant functions in controlling the balance of SST and PV CINs generation, whereas in postnatal maturing CINs *Mafb* and *c-Maf* have opposite functions in controlling CIN physiology. Ongoing studies are aimed at elucidating the molecular mechanisms underlying these overlapping and distinct functions of *Mafb* and *c-Maf.*

## STAR★METHODS

### CONTACT FOR REAGENTS AND RESOURCE SHARING

Further information and requests for resources and reagents should be directed to and will be fulfilled by the Lead Contact, John Rubenstein (john.rubenstein@ucsf.edu).

### EXPERIMENTAL MODEL AND SUBJECT DETAILS

#### Animals

All procedures and animal care were approved and performed in accordance with the University of California San Francisco Laboratory Animal Research Center (LARC) guidelines. All mice strains have been previously published: *Ai14* Cre-reporter (Madisen et al., 2010), *Nkx2.1-Cre* (Xu et al., 2008), *Mafb* flox (Yu et al., 2013), *c-Maf* flox (Wende et al., 2012), *799-CreER* (Silberberg et al., 2016) and *Sst-IRES-Cre* (Taniguchi et al., 2011). Mice were backcrossed onto a CD-1 background before analyses. For timed pregnancies, noon on the day of the vaginal plug was counted as embryonic day 0.5. Mouse crosses generated both pure *Mafb* and *c-Maf* single mutants and those that were hemizygous for the other gene. We did not observe gross phenotypic differences between mice with or without the additional hemizygous allele, and these were combined together for analysis. For *799-CreER* experiments, tamoxifen (5mg/40 g) was administered intraperitoneally to activate the *CreER*, at embryonic day 11.5. All analyses included both males and females.

For the genotype information regarding the mice used for each analysis, please see the table below. The sensitized single mutants carry a hemizygous allele for the other *Maf* gene.

**Table:** 

Figure(s)	Genotype information			
	Control	*Mafb* cKO	*c-Maf* cKO	cDKO
Figures 1 and S3	WTs	Pure *Mafb* cKOs	Pure *c-Maf* cKOs	n.a
Figure 2 Neocortical analysis	A mixture of WTs and double heterozygous controls	A mixture of pure *Mafb* cKOs and sensitized *Mafb* cKOs	*c-Maf* cKOs	cDKOs
Figures 3, 4, 5, S4, and S5	A mixture of WTs and double heterozygous controls	n.a	n.a	cDKOs
Figure 6 synapse analysis; Figures S2 and S6 morphology analysis	A mixture of WTs and double heterozygous controls	A mixture of pure *Mafb* cKOs and sensitized *Mafb* cKOs	A mixture of pure *c-Maf* cKOs and sensitized *c-Mafb* cKOs	cDKOs
Figures 6 and 7 electrophysiology	WTs	Pure *Mafb* cKOs	Pure *c-Maf* cKOs	cDKOs
Figure S1	WTs	Pure *Mafb* cKO	Pure *c-Maf* cKO	n.a

### METHODS DETAILS

#### EdU injections and analysis

Pregnant mice were pulsed with 5-Ethynyl-2′-Deoxyuridine (EdU), 10mg/ml (Thermo Fisher Scientific E10187), at a dose of 50mg EdU/kg body weight. For MGE S-phase progenitor quantification (EdU pulse), E12.5-E13.5 mice were sacrificed 30 minutes after EdU injection and collected in ice-cold PFA/PBS. For MAFB/c-MAF and EdU Fig.colabeling experiments, E12.5 embryos were harvested 1 hr after EdU injection. For pulse-chase experiments, mice or progeny were sacrificed at E15.5 or P35. Embryonic and postnatal brains were collected and fixed overnight in 4% PFA at 4°C, and then sunk in 30% sucrose before embedding in OCT. EdU^+^ cells were visualized using standard procedures in the Clik-iT EdU plus kit (Thermo Fisher Scientific C10340) that were co-stained with DAPI. For pulse-chase experiments, the same parameters were used, and the only differential factor was the time needed before analysis.

#### Immunofluorescence/Immunohistochemistry

All tissues were fixed with 4% PFA 1-2 hr (for > P7 tissues) or overnight (< P7 tissues), followed by 30% sucrose cryoprotection. P7, P16 and P35 fixed tissues were sectioned coronally at 40 μm and stained free-floating. All embryonic ages and P0 fixed tissue were sectioned coronally, at 20 μm, and stained on glass slides. P40 transplant tissue was sectioned coronally at 25 μm and stained on glass slides. Immunofluorescent labeling was performed with the following primary antibodies: rabbit anti-Mafb (Sigma HPA005653; 1:500), rabbit anti-c-MAF (Santa Cruz Biotechnology sc-7866; 1:500), rabbit anti-parvalbumin (Swant PV25; 1:200), rat anti-somatostatin (Millipore MAB354; 1:200), goat anti-somatostatin (Santa Cruz Biotechnology sc-7819; 1:100), rabbit anti-VIP (Immunostar 20077; 1:100), rabbit anti-nNOS (Life Technologies 61-7000; 1:200), goat anti-SP8 (Santa Cruz Biotechnology sc-104661; 1:100), mouse anti-Tuj1 (Covance MMS-435P; 1:500), goat anti-MCM2 (Santa Cruz Biotechnology sc-9839; 1:200 (Maslov, 2004), rabbit anti-KI67(Abcam ab15580; 1:500), mouse anti-KI67 (BD Biosciences 550609 ; 1:200), rabbit anti-VGLUT1 (synaptic system 135303; 1:500), rabbit anti-VGAT (synaptic system 131002; 1:200), mouse anti-GEPHRIN (synaptic system 147011; 1:500) and mouse anti-PSD95 (NeuroMab 75-028, clone ID K28/43; 1:500). The appropriate 488, 594 or 647 Alexa-conjugated secondary antibodies (1:500) were from Life Technologies. All primary and secondary antibodies were diluted in PBS containing 2.5% BSA and 0.3% Triton X-100. Sections were coverslipped with Vectashield containing DAPI (Vector labs).

#### *In situ* hybridization

*In situ* hyrbidization was performed as previously described (McKinsey et al., 2013). Probes included *CoupTF2* (M. Tsai), *Cxcr7* (ATCC MGC-18378), *CyclinD2* (A. Malamacci), *Lhx6* (V. Pachnis), *Mafb* (J. Rubenstein), *c-Maf* (J. Rubenstein), *Nkx2.1* (J. Rubenstein), *Sox6* (Open Biosystems, Clone #5269193), *Sp8* (C. Belmonte), Sst (T. Lufkin) were used.

To generate the *Mafb* DNA vector and riboprobe, *Mafb* cDNA was PCR amplified from mouse genomic DNA (mixed CD-1; C57BL6J) using the following primers:

5′ GAGAGTCGACATGGCCGCGGAGCTGAGC

3′ ATATGAGCTCTCACAGAAAGAACTCGGG.

SalI and SacI restriction enzymes sites were introduced (underlined). Next, the *Mafb* PCR product and the vector, pB3.p11 (Addgene # 69577), were digested with SalI and SacI, and then ligated. The *Mafb* RNA anti-sense probe was generated by T3 RNA polymerase from a SalI linearized vector, with the size of the probe ~1kb.

#### MGE transplantation

A detailed protocol for this procedure is available in a methods format (Vogt et al., 2015). We bred male mice homozygous for *Ai14* and were either WT (control), *Mafb^Flox/Flox^*, *c-Maf^Flox/Flox^* or *Mafb^Flox/Flox^*; *c-Maf^Flox/Flox^* to females that were *Ai14* negative but WT or homozygous for each of the corresponding male alleles, respectively. These crosses yielded embryos that were either WT or homozygous for each of the *Maf* alleles. The embryos were collected at E13.5, dissociated and then transduced with a *DlxI12b-Cre* lentivirus(Vogt et al., 2015, 2018) in DMEM supplemented with 10% Fetal Bovine Serum (FBS) at 37°C and at pH ~7.2 for 30 minutes. This virus deletes the *Maf* genes and activates tdTomato expression from the *Ai14* allele. The cells were then washed several times with DMEM/FBS pelleted. Next, the cells were loaded into the front of a beveled glass needle. P1 WT pups were anesthetized on ice and injected with ~300 nL of cells over 3-5 sites in the right hemisphere. Pups were warmed until able to move and then put back with their mom. They were aged to 40 days and then perfused. Cells were analyzed as described above.

#### Primary neuronal culture for analysis of dendritic arborization (Sholl) and synapses using neonatal cortex

Briefly, we bred *Mafb*^Flox/Flox^, *c-Maf^Flox/Flox^, Ai14f^Flox/Flox^* females with *Mafb^Flox/+^, c-Maf^Flox/+^, Nkx2-1 Cre*^+^ males to generate P0 *Mafb* cKO, *c-Maf* cKO and cDKOs. Control P0 animals were generated either through the same crossing or through *Ai14f^Flox/Flox^* females bred with *Nkx2-1 Cre*^+^ males. tdTomato^+^ P0 pups were pre-screened using fluorescence dissection microscope. We also collected some tdTomato^−^ P0 pups for cell preparation. Cortical tissues were dissected in cold EBSS, followed by trypsin (Thermo Fisher Scientific 25200056) treatment for 15 minutes at 37°C. Trypsinization was inhibited using 10% FBS containing DMEM. Cells were washed once with DMEM, then resuspended in 10% FBS containing Neuralbasal-A medium (Thermo Fisher Scientific 12348017) with B27 (Thermo Fisher Scientific 17504044). Cell density was quantified using hemocytometer. tdTomato^+^ cell preparation was diluted using tdTomato^−^ P0 cell preparation roughly at a ratio of 1:10. Cells were plated in poly-D-lysine and laminin coated coverslips (Corning 08-774-385) preloaded in 24-well plates and cultured in 37°C incubator for 14 days. Serum free Neuralbasal-A medium with B27 and Glutamax (Thermo Fisher Scientific 35050061) was used to maintain the cell growth. At day *in vitro* 14, cell culture medium was removed and replaced with freshly made 4% PFA for 15min fixation. PFA was washed off several times with 1× PBS followed by regular immunostaining protocol for synapse labeling. (1) For synapse quantification, ≥ 3 animals and 25-40 proximal dendrites were analyzed per genotype. Colocalizations were scored if pre- and post-synaptic puncta along tdTomato-labeled proximal dendrites (within 15um from soma) were fully overlapped. We performed a One way ANOVA followed by Turkey’s multiple comparisons test. Differences were regarded significant if p < 0.05. (2) For Sholl analysis, ≥ 3 animals and 15-20 neurons were analyzed per genotype. Images were processed and analyzed using FIJI software based on previously described protocol (Ferreira et al., 2014; Martin et al., 2018).

#### Image acquisition and processing

1. Immunohistochemistry images were taken using a CoolSNAP EZ Turbo 1394 digital camera (Photometrics) mounted on a Nikon Eclipse 80i microscope (Nikon Instruments) using NIS Elements acquisition software (Nikon). Images for proliferative markers were taken using Nikon Ti inverted fluorescence microscope with a CSU-W1 large field of view confocal microscope that had 20X and 60X oil objectives to visualize marker colocalization. Brightness and contrast were adjusted, and images merged using FIJI software.
2. Sholl analysis and excitatory synapse images were taken using the Nikon Ti inverted fluorescence microscope with CSU-W1 large field of view confocal. Images for Sholl analysis were taken using 40X oil objective while images for excitatory synapse quantification were taken using a 60X oil objective. Open source micromanager 2.0 beta was used to acquire images. Brightness/contrast adjustment, z stack image and binary image processing and Sholl analysis were all conducted using the image calculator and Sholl analysis plugins in FIJI software.

#### Electrophysiology

##### Slice preparation

Mice were euthanized with 4% isoflurane, perfused with ice-cold sucrose cutting solution containing 234 mM sucrose, 2.5 mM KCl, 1.25 mM NaH2PO4, 10 mM MgSO4, 0.5 mM CaCl2, 26 mM NaHCO3, and 11 mM glucose, equilibrated with 95% O2 and 5% CO2, pH 7.4, and decapitated. We prepared 250 μm-thick horizontal thalamic slices containing Somatosensory Barrel Cortex with a Leica VT1200 microtome (Leica Microsystems). We incubated the slices, initially at 32°C for 1 h and then at 24–26°C, in artificial cerebrospinal fluid (ACSF) containing 126 mM NaCl, 2.5 mM KCl, 1.25 mM NaH2PO4, 2 mM MgCl2, 2 mM CaCl2, 26 mM NaHCO3, and 10 mM glucose, equilibrated with 95% O2 and 5% CO2, pH 7.4 as described in (Clemente-Perez et al., 2017; Paz et al., 2011,^2013^).

##### Whole-cell patch-clamp electrophysiology

Recordings were performed as previously described (Clemente-Perez et al., 2017; Paz et al., 2011, 2013). We visually identified interneurons originating from the MGE based on their expression of tdTomato. Neurons were identified by differential contrast optics with a Zeiss (Oberkochen) Axioskop microscope and an infrared video camera. Recording electrodes made of borosilicate glass had a resistance of 2.5–4 MΩ when filled with intracellular solution. Access resistance was monitored in all the recordings, and cells were included for analysis only if the access resistance was < 25 MΩ. The access resistance was similar in all recordings (p > 0.5), suggesting that differences between genotypes were not due to the quality of the whole-cell patch-clamp recording. Spontaneous excitatory post-synaptic currents (sEPSCs) were recorded in the presence of picrotoxin (50 μM, Tocris). For sEPSCs and current-clamp recordings, the internal solution contained 120 mM potassium gluconate, 11 mM KCl, 1 mM MgCl2, 1 mM CaCl2, 10 mM HEPES, and 1 mM EGTA, pH adjusted to 7.4 with KOH (290 mOsm). Tetrodotoxin 1 μM was added to the extracellular solution for miniature EPSC (mEPSC) recordings. The experiments were performed by blinded observers. To test for differences in the sEPSC/mEPSC amplitude, frequency, and decay tau dataset across genotypes (comparison between group means), we performed a One Way ANOVA Kruskal-Wallis followed by Dunn’s multiple comparisons test. Differences were regarded significant if p < 0.05.

*F-I* plots were generated using a custom MATLAB code. To test for differences in the F-I dataset across genotypes (comparison between group means), we performed a regular two-way ANOVA followed by Tukey’s post hoc test for multiple comparisons. Differences were regarded significant if p < 0.05. For statistical analysis, we included only the current pulses that were presented to all genotypes (within cell type). In addition, we only included cells which were recorded at at least two of the included current pulses. For FS cells, we analyzed responses at current pulses of 20, 60, 100, 140, 180, 220, and 260 pA. For RS cells, we analyzed responses at current pulses of 20, 60, and 100 pA.

##### Extracellular cortical local field potential recordings

Coronal slices (400 μm) containing Somatosensory Barrel Cortex were placed in a humidified, oxygenated interface chamber and perfused at a rate of 2 mL/min at 34°C with oxygenated ACSF prepared as described above and supplemented with 300 μM glutamine for cellular metabolic support (Clemente-Perez et al., 2017; Paz et al., 2011, 2013). Extracellular LFP recordings were obtained with a 16-channel multi-electrode array (Neuronexus) placed in the Somatosensory Barrel Cortex. The signals were sampled at 24.414 kHz. Signals were amplified at 10,000× and band-pass filtered between 100 Hz and 6 kHz using the RZ5 from Tucker-Davis Technologies (TDT). We delivered electrical stimuli to the internal capsule with a concentric bipolar tungsten electrode (50–100 kΩ, FHC) and recorded the evoked local field potential (LFP) responses. Stimulations were repeated 10–30 times in a single recording and an average LFP was calculated. To assess the cortical network excitability, electrical stimulation was delivered in the internal capsule in increasing amplitudes starting from 10 μA to 500 μA. The experiments were performed by blinded observers.

### QUANTIFICATION AND STATISTICAL ANALYSIS

All bar graphs were shown as mean ± SEM. All statistical analyses were performed using Graphpad Prism (version 7) or R-Project3.1, and a p value of < 0.05 was considered significant. The specific n for each experiment as well as the post hoc test corrected p values, can be found in the Results section, in the Figure legends or in supplementary tables.

1. For all cell counts performed for immunohistochemistry and *in situ* hybridization, we used the cell counter plug-in in FIJI software. For statistical analyses, we used two methods depending on whether data were parametric or nonparametric in distribution. For parametric data, we utilized a two-tailed t test or one-way ANOVA, followed by a Tukey’s post hoc test, depending on the number of samples being compared. For any data that was normalized (i.e., cell transplants normalized to total cells transplanted or cell fate analyses where proportions were calculated) we used the non-parametric Chi-square test.
2. For LFP analysis: From an average of responses for each slice and intensity, we calculated line length L as the sum of the absolute differences between successive points in a 30 ms signal s just after the stimulation as L = Σi = 1N|s[i−1]−s[i]|, similar to (Esteller et al., 2001). The average amplitude was calculated as the absolute difference between the minimum and maximum of the signal as |max(s)−min(s)| from a similar window. Current source densities (CSDs) were computed as the negative of the second difference across channels divided by the square of a nominal spatial differentiation grid g as: CSD = −d(d(S))/g, where S is a matrix with each row a signal for an individual channel like,

S = (s1, 1 s1,2 s1,… s1, N s2, 1 s2,2 s2,… s2, N s…,1 s…,2 s1,… s…,N s16, 1 s16, 2 s16,… s16, N), and d(·) is the first difference operator computed by column. For more theory see (Hodgkin and Huxley, 1952).

Numerical values are given as mean ± SEM unless stated otherwise. 6 Controls, 7 *Mafb* cKOs, 6 *c-Maf* cKOs and 3 cDKOs were used. For statistical analyses, we used parametric and nonparametric tests. We assessed statistical significance, as appropriate, by performing two-way ANOVA and the Kolmogoroff-Smirnoff test using R-Project 3.1.

## Supplementary Material

Table S1

2

## Figures and Tables

**Table:** KEY RESOURCES TABLE

REAGENT or RESOURCE	SOURCE	IDENTIFIER
Antibodies		
Rabbit anti-PARVALBUMIN antibody	Swant	PV27; RRID:AB_2631173
Rat anti-SOMATOSTATIN antibody	Millipore Sigma	MAB354; RRID:AB_2255365
Rabbit anti-MAFB antibody	Millipore Sigma	HPA005653; RRID:AB_1079293
Rabbit anti-c-MAF antibody	Santa Cruz Biotechnology	sc-7866; RRID:AB_638562
Goat anti-SOMATOSTATIN antibody	Santa Cruz Biotechnology	sc-7819; RRID:AB_2302603
Rabbit anti-VIP antibody	Immunostar	20077; RRID:AB_1073072
Rabbit anti-nNOS antibody	Life Technologies	61-7000; RRID:AB_2313734
Goat anti-SP8 antibody	Santa Cruz Biotechnology	sc-104661; RRID:AB_2194626
Mouse anti-TUJ1 antibody	Covance	MMS-435P; RRID:AB_2313773
Goat anti-MCM2 antibody	Santa Cruz Biotechnology	sc-9839; RRID:AB_648841
Rabbit anti-KI67 antibody	Abcam	ab15580; RRID:AB_443209
Mouse anti-KI67 antibody	BD Biosciences	550609; RRID:AB_393778
Rabbit anti-VGLUT1 antibody	synaptic system	135303; RRID:AB_887875
Mouse anti-PSD95 antibody	NeuroMab	75-028, clone ID K28/43; RRID:AB_2292909
Bacterial and Virus Strains		
*pLenti_Dlxi12b-MCS-IRES-Cre* lentivirus	Vogt et al., 2018	N/A
One shot DH5alpha STBL3 bacteria	Thermo Fisher	C737303
Chemicals, Peptides, and Recombinant Proteins		
Trypsin	Thermo Fisher Scientific	25200056
Neuralbasal-A medium	Thermo Fisher Scientific	12348017
B27 supplement	Thermo Fisher Scientific	17504044
Glutamax	Thermo Fisher Scientific	35050061
Penicillin-Streptomycin	Hyclone	SV30010
poly-D-lysine and laminin coated coverslips	Corning	08-774-385
EdU	Invitrogen	E10187
Click-iT Plus EdU Alexa Fluor 647 Imaging Kit	Invitrogen	C10340
picrotoxin	Tocris	1128
Deposited Data		
GSE94641 (MGE cells)	Chen et al., 2017	https://doi.org/10.1038/srep45656.
Experimental Models: Organisms/Strains		
*Nkx2.1-Cre* mouse line	Xu et al., 2008	Jax labs #008661
*SST-IRES-Cre* mouse line	Taniguchi et al., 2011	Jax labs #013044
*Ai14* (Cre-dependent reporter) mouse line	Madisen et al., 2010	Jax labs #007908
*Mafb*^*Flox*^ mouse line	Yu et al., 2013	N/A
*c-Maf*^*Flox*^ mouse line	Wende et al., 2012	N/A
Oligonucleotides		
5′ primer for Mafb riboprobe cloning: GAGAGTCGACATGGCCGCGGAGCTGAGC	ELIM Biosciences	N/A
3′ primer for Mafb riboprobe cloning: ATATGAGCTCTCACAGAAAGAACTCGGG	ELIM Biosciences	N/A
Recombinant DNA		
pB3.p11 (for synthesize Mafb riboprobe)	Addgene	C737303
Sox6 *In situ* probe	Open Biosystems	Clone #5269193
Cxcr7 *In situ* probe	ATCC MGC	18378
Software and Algorithms		
NIS Elements software	Nikon	N/A
Prism v.7	GraphPad	N/A
Image-J software	NIH	N/A
Micromanager	https://micro-manager.org/	N/A
FIJI	https://imagej.net/Fiji	N/A
MATLAB	https://www.mathworks.com/products/matlab.html	N/A
R-Project 3.1	https://www.r-project.org/	N/A
Other		
CoolSNAP EZ Turbo 1394 digital camera	Photometrics	N/A
Nikon Eclipse 80i microscope	Nikon Instruments	N/A
Nikon Ti inverted fluorescence microscope	Nikon Instruments	N/A
Leica VT1200 microtome	Leica Microsystems	N/A
Zeiss (Oberkochen) Axioskop microscope	Zeiss	N/A
16-channel multi-electrode	Neuronexus	N/A
RZ5 signal amplifier	Tucker-Davis Technologies	N/A
BioRender	https://biorender.com	N/A

## Highlights

- *Mafb* and *c-Maf* are expressed in MGE-derived CINs but not projection neurons
- *Mafb* and *c-Maf* control the numbers of MGE-lineage CINs
- Loss of *Mafb* and *c-Maf* leads to increased SST^+^ CIN perinatally
- *Mafb* and *c-Maf* control CIN synaptic maturation antagonistically
