# Supplementary material for: Mafb and c-Maf Have Prenatal Compensatory and Postnatal Antagonistic Roles in Cortical Interneuron Fate and Function
Source: Cell Rep. Author manuscript; Available in PMC 2019 Jul 1. (PMC6602795; doi:10.1016/j.celrep.2019.01.031)
Supplement: 2 [file NIHMS1024767-supplement-2.pdf]

Cell Reports, Volume *26*

Supplemental Information

Mafb and *c-Maf* Have Prenatal Compensatory and Postnatal Antagonistic Roles in Cortical Interneuron Fate and Function

Emily Ling-Lin Pai, Daniel Vogt, Alexandra Clemente-Perez, Gabriel L. McKinsey, Frances S. Cho, Jia Sheng Hu, Matt Wimer, Anirban Paul, Siavash Fazel Darbandi, Ramon Pla, Tomasz J. Nowakowski, Lisa V. Goodrich, Jeanne T. Paz, and John L.R. Rubenstein

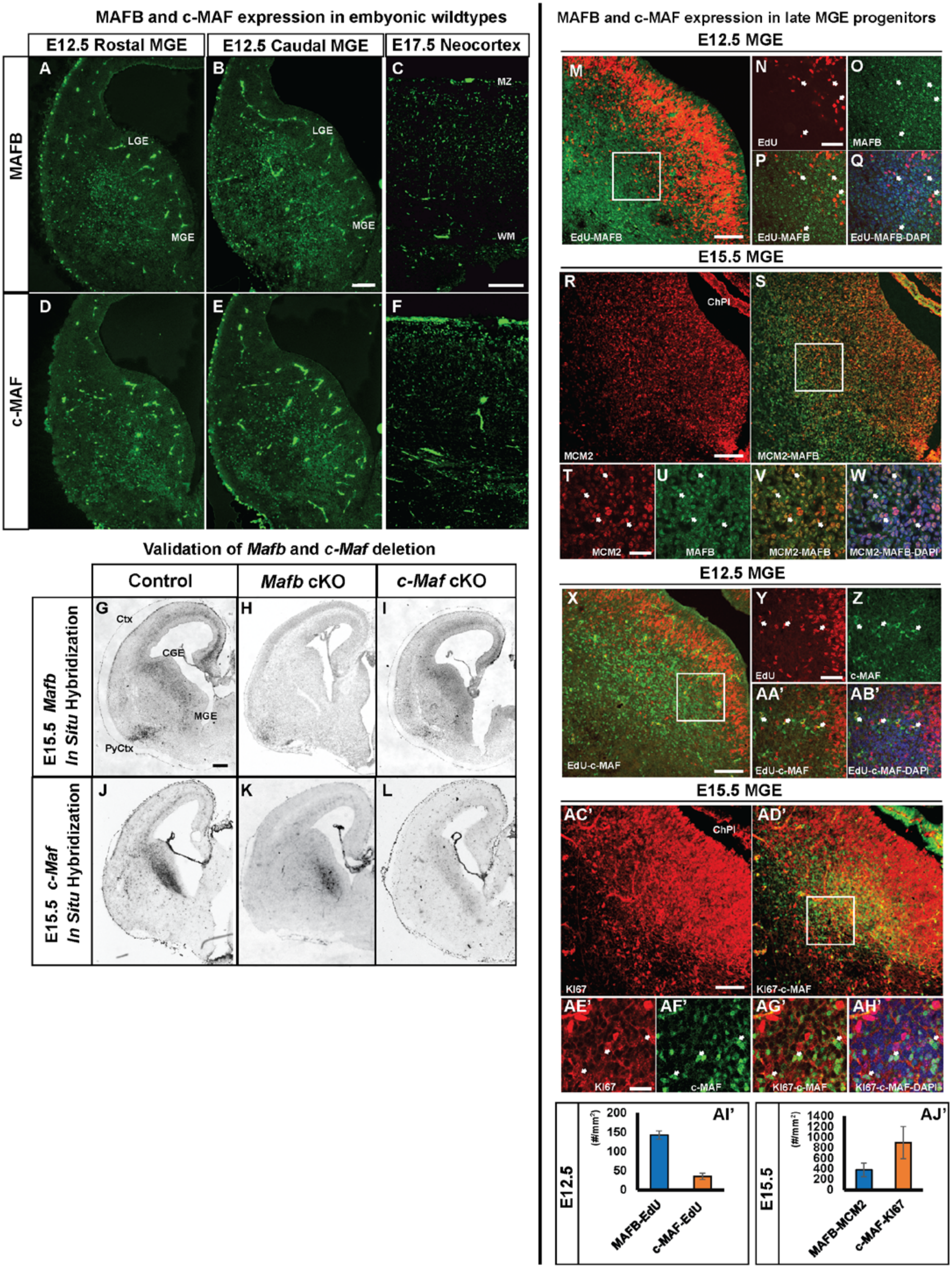

MAFB and c-MAF expression in embyonic wildtypes
E12.5 Rostal MGE
E12.5 Caudal MGE
E17.5 Neocortex
MAFB
c-MAF
A
B
C
D
E
F
LGE
MGE
MZ
WM
Validation of Mafb and c-Maf deletion
Control
Mafb cKO
c-Maf cKO
E15.5 Mafb In Situ Hybridization
E15.5 c-Maf In Situ Hybridization
G
H
I
J
K
L
Ctx
CGE
PyCtx
MAFB and c-MAF expression in late MGE progenitors
E12.5 MGE
M
N
O
P
Q
EdU
MAFB
EdU-MAFB
EdU-MAFB-DAPI
E15.5 MGE
R
S
ChPl
MCM2
MCM2-MAFB
T
U
V
W
MCM2-MAFB-DAPI
E12.5 MGE
X
Y
Z
AA'
AB'
c-MAF
EdU-c-MAF
EdU-c-MAF-DAPI
E15.5 MGE
AC'
AD'
KI67
KI67-c-MAF
AE'
AF'
AG'
AH'
KI67-c-MAF-DAPI
AI'
AJ'
E12.5
E15.5
(#/mm²)
200
150
100
50
0
1400
1200
1000
800
600
400
200
MAFB-EdU
c-MAF-EdU
MAFB-MCM2
c-MAF-KI67

Figure S1. (A-F) MAFB and C-MAF expression at E12.5 and E17.5; (G-L): Effect of *Maf* mutations on the expression of *Mafb* and *c-Maf* RNA; (M-AJ'): MAFB and C-MAF expression in dividing MGE cells. *Related to Figure 1 and Table S1.*

(A-F) Immunofluorescent images from E12.5 basal ganglia and E17.5 neocortices showing MAFB and c-MAF positive cells. **(G-L)** Effect of *Mafb* and *c-Maf* deletion on RNA expression in the *Mafb* cKO/*c-Maf* cKOs at E15.5, compared to control brain. **(M-Q)** Immunofluorescent images from E12.5 MGE that show co-localization of MAFB and EdU. EdU was injected when embryos were E12.5 and was incubated for an hour to label the majority of VZ progenitors and some SVZ progenitors. **(R-W)** Immunofluorescent images from E15.5 MGE that show co-localization of MAFB and MCM2, a pan progenitor marker. **(X-AB')** Immunofluorescent images from E12.5 MGE that show co-localization of c-MAF and EdU. **(AC'-AH')** Immunofluorescent images from E15.5 MGE that show co-localization of c-MAF and KI67, another pan progenitor marker. Arrowheads point to examples of cells that are $MAFB^+$ or $c\text{-}MAF^+$ progenitors. Boxed regions indicate the approximate regions used for high magnification imaging. **(AI')** Quantification of cell density that are co-expressing MAFB and EdU or C-MAF and EdU at E12.5. Data are shown as mean ± SEM. Six technological replicates were used for analysis. **(AJ')** Quantification of cell density that are co-expressing MAFB and MCM2 or c-MAF and KI67 at E15.5. Data was shown in mean ± SEM. Six technological replicates were used for analysis. Scale bar in **(B)** and **(G)** = 250um. Scale bar in **(C)** = 100um. Scale bar in **(M, R, X and AC')** = 100um. Scale bar in **(N, T and Y)** = 50 um. Scale bar in **(AE')** = 25um.

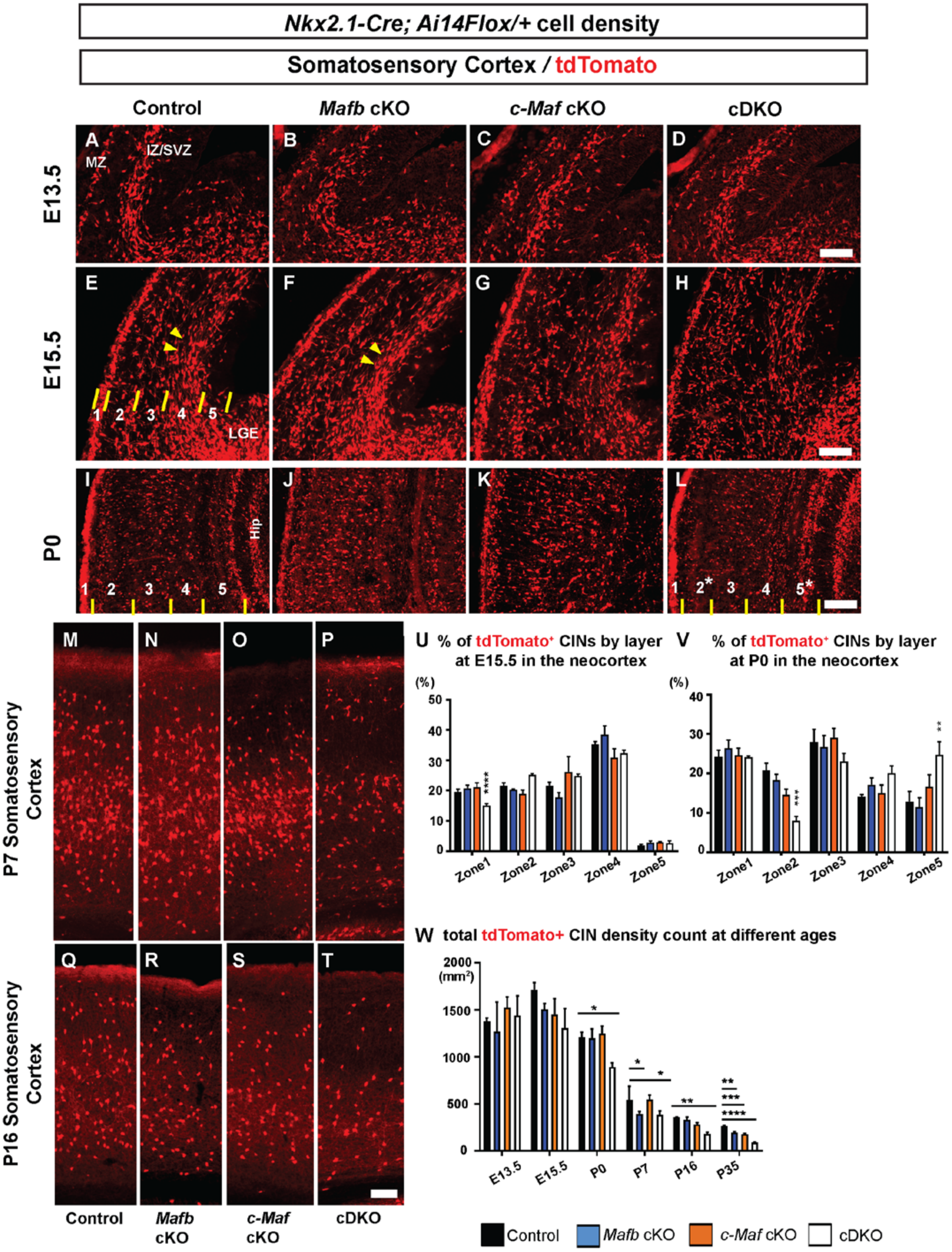

Nkx2.1-Cre; Ai14Flox/+ cell density
Somatosensory Cortex / tdTomato
Control
Mafb cKO
c-Maf cKO
cDKO
E13.5
E15.5
P0
MZ
IZ/SVZ
LGE
Hip
P7 Somatosensory Cortex
P16 Somatosensory Cortex
U % of tdTomato+ CINs by layer at E15.5 in the neocortex
V % of tdTomato+ CINs by layer at P0 in the neocortex
Zone1
Zone2
Zone3
Zone4
Zone5
W total tdTomato+ CIN density count at different ages
(mm2)
E13.5
E15.5
P0
P7
P16
P35

Figure S2. ***Mafb/ c-Maf*** **cKO and cDKOs have gradual reduction of CINs by P35.** *Related to Figure 2 and Table S2.*

(A-T) Immunofluorescent images from E13.5/ E15.5/ P0 neocortices and P7/ P16 somatosensory cortices show native $tdTomato^+$ CIN distribution. **(U)** Quantification of the proportion of $tdTomato^+$ cells by regions at E15.5 neocortex. Zone 1= marginal zone; Zone 2 = cortical plate and subplate; Zone 3 = intermediate zone; Zone 4 = deep migration/subventricular zone; Zone 5= ventricular zone. **(V)** Quantification of the proportion of $tdTomato^+$ cells by regions at P0 neocortex. Cortex was binned into 5 zones roughly by equal distance, but zone 1 was focused on the marginal zone. **(W)** Quantification of the number of $tdTomato^+$ cells per mm^2 in the neocortices or somatosensory cortices. Data are expressed as the mean ± SEM. *$p < 0.05$, **$p < 0.01$, ***$p < 0.001$. (n) = 3-4 for all groups. Scale bar in (**D, H, L, P and T**) = 100 μm. The P35 data is the same as shown in Fig. 2.

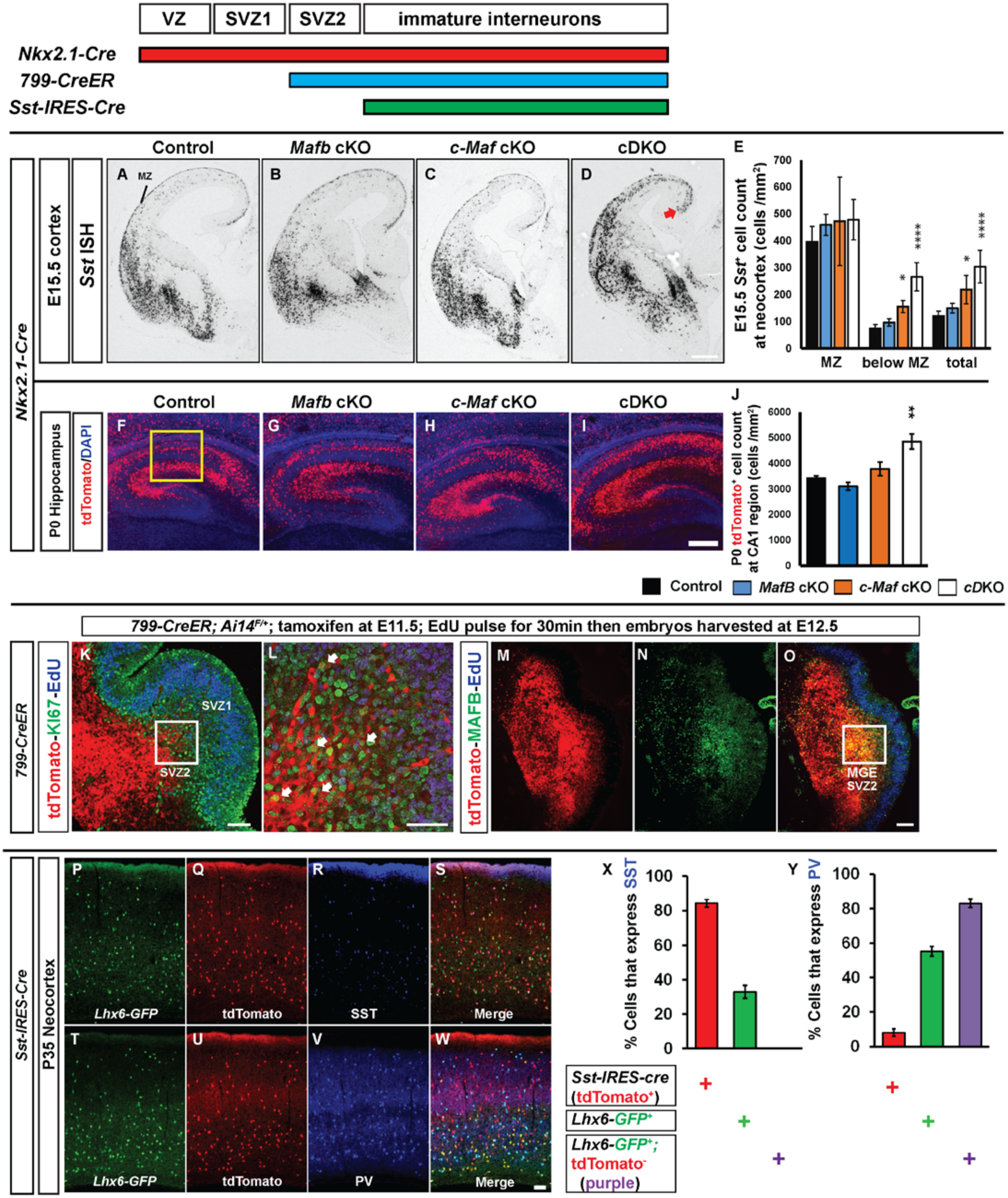

VZ
SVZ1
SVZ2
immature interneurons
Nkx2.1-Cre
799-CreER
Sst-IRES-Cre
Control
Mafb cKO
c-Maf cKO
cDKO
E15.5 cortex
Sst ISH
A
MZ
B
C
D
E
E15.5 Sst⁺ cell count at neocortex (cells /mm²)
700
600
500
400
300
200
100
0
MZ
below MZ
total
Nkx2.1-Cre
P0 Hippocampus
tdTomato/DAPI
F
G
H
I
J
P0 tdTomato⁺ cell count at CA1 region (cells /mm²)
6000
5000
4000
3000
2000
1000
0
Control
MafB cKO
c-Maf cKO
cDKO
799-CreER; Ai14F/+; tamoxifen at E11.5; EdU pulse for 30min then embryos harvested at E12.5
799-CreER
tdTomato-KI67-EdU
K
SVZ1
SVZ2
L
tdTomato-MAFB-EdU
M
N
O
MGE
SVZ2
Sst-IRES-Cre
P35 Neocortex
P
Lhx6-GFP
Q
tdTomato
R
SST
S
Merge
T
Lhx6-GFP
U
tdTomato
V
PV
W
Merge
X
% Cells that express SST
100
80
60
40
20
0
Y
% Cells that express PV
100
80
60
40
20
0
Sst-IRES-cre (tdTomato⁺)
Lhx6-GFP⁺
Lhx6-GFP⁺; tdTomato⁻ (purple)

Figure S3. (A-E): Effect of *Maf* single and double conditional mutants *(Nkx2.1-Cre)* on the expression of *Sst* RNA at E15.5; (F-J): Effect of *Maf* single and double conditional mutants *(Nkx2.1-Cre)* on the numbers of hippocampal tdTomato$^+$ interneurons at P0; (K-O) *799-CreER* activity initiates in the late SVZ (SVZ2) of MGE; (P-Y) Fate mapping of *Sst-IRES-Cre* lineage CINs in the adult CINs. *Related to Figure 2 and Figure 4.*

Top: The schema shows the cell types in which each *Cre* line initiates recombination.

***Nkx2.1-Cre* data: (A-D)** *Sst in situ* hybridization at E15.5 in control, *Mafb* cKO, *c-Maf* cKO and cDKO. **(E)** Quantification of *Sst*$^+$ CINs per mm^2 by region in the neocortex in 4 different *Maf* genotypes. (n)=3-4 for all groups. Note that in the *c-Maf* cKOs, there was no reduction in the *Sst*$^+$ CIN density count. **(F-I)** Immunofluorescent images of the P0 hippocampus of the 4 different *Maf* genotypes. Boxed region in **(F)** highlights the CA1 region used for tdTomato$^+$ CIN quantification for each genotype. **(J)** Quantification of tdTomato$^+$ CINs per mm^2 in the CA1 region; (n)=4 for all *Maf* genotypes.

***799-CreER* data: (K)** Immunofluorescent images from E12.5 MGE that show co-staining of tdTomato (*799-CreER* lineage), EdU and KI67. **(L)** Higher magnification views (imaged from the boxed region in K) showing colocalization of tdTomato and KI67 in the SVZ2 of the MGE. **(M-O)** Immunofluorescent images from E12.5 embryonic basal ganglia that show co-staining of tdTomato, EdU and MAFB. Note the boxed region in **(L)** that show colocalization of tdTomato and MAFB, providing evidence that *799-CreER* activity and MAFB expression both initiate in SVZ2 (late SVZ) of the MGE.

***Sst-IRES-Cre* data: (P-S)** Immunofluorescent images from P35 neocortex that show the overlay of SST$^+$ CINs with the *Sst-IRES-Cre* lineage (tdTomato$^+$) and Lhx6-GFP$^+$ cells (mostly MGE-derived CINs). **(T-W)** Immunofluorescent images from P35 neocortex that show the overlay of PV$^+$ CINs with the *Sst-IRES-Cre* lineage (tdTomato$^+$) and Lhx6-GFP$^+$ cells. **(X)** Quantification of the percentage of tdTomato$^+$ CINs, Lhx6-GFP$^+$ CINs or tdTomato$^-$;GFP$^+$ CINs that express SST. 80% of tdTomato$^+$ cells (*Sst-IRES-Cre*-lineage) were SST$^+$. ~35% of Lhx6-GFP$^+$ CINs were SST$^+$. None of the tdTomato$^-$/GFP$^+$ cells were SST$^+$. **(Y)** Quantification of the percentage of tdTomato$^+$ CINs, Lhx6-GFP$^+$ CINs or tdTomato$^-$;GFP$^+$ CINs that express PV. ~8% of tdTomato$^+$ cells (*Sst-IRES-Cre*-lineage) were PV$^+$. ~55% of *Lhx6-GFP*$^+$ CINs were PV$^+$. ~85% of the tdTomato$^-$/GFP$^+$ cells were PV$^+$. Together, this data provide evidence that most of the CINs derived from the *Sst-IRES-Cre* lineage are SST$^+$. Data are expressed as the mean ± SEM. *p < 0.05, **p < 0.01, ****p < 0.0001. Scale bars in **(I, K and W)** =100um; Scale bar in **(L)** = 50 μm; Scale bar in **(D and O)** = 200 um; Abbreviations: VZ: ventricular zone, SVZ1: early subventricular zone, SVZ2: late subventricular zone, MZ: marginal zone.

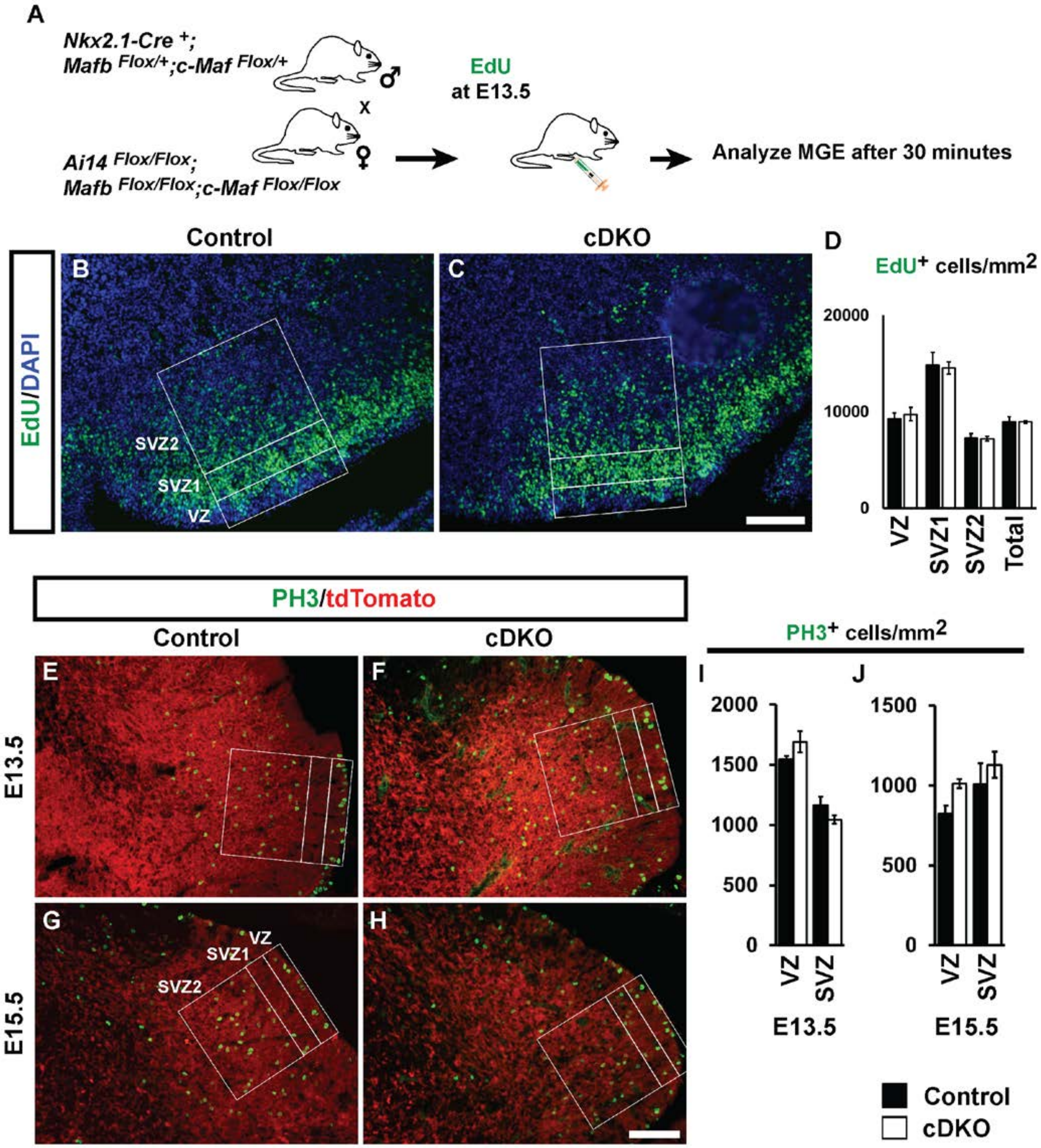

Figure S4. MGE progenitors show no differences in proliferation indices in *Maf* cDKOs. *Related to Figure 2, Figure 3, Figure 4 and Figure 5.*

(A) Schema depicting the EdU 30-minute pulse assay. Briefly, EdU was injected into pregnant females when embryos were E13.5 and assessed after 30 minutes. Immunofluorescent images show the MGE co-stained with EdU and DAPI **(B-C)**. **(D)** Quantification of EdU$^+$ progenitors per mm^2 by region. Immunofluorescent images from either E13.5 **(E and F)** or E15.5 **(G and H)** MGEs that were co-labeled for tdTomato and PH3. Quantification of the numbers of PH3$^+$ cells/area were calculated at E13.5 **(I)** and E15.5 **(J)** for both VZ and SVZ regions. Data are expressed as the mean ± SEM. (n)=3-4 for all groups. Student t-test was done but found no significant changes. Scale bars in **(C and H)** = 100 µm. Abbreviations: VZ: ventricular zone, SVZ: subventricular zone

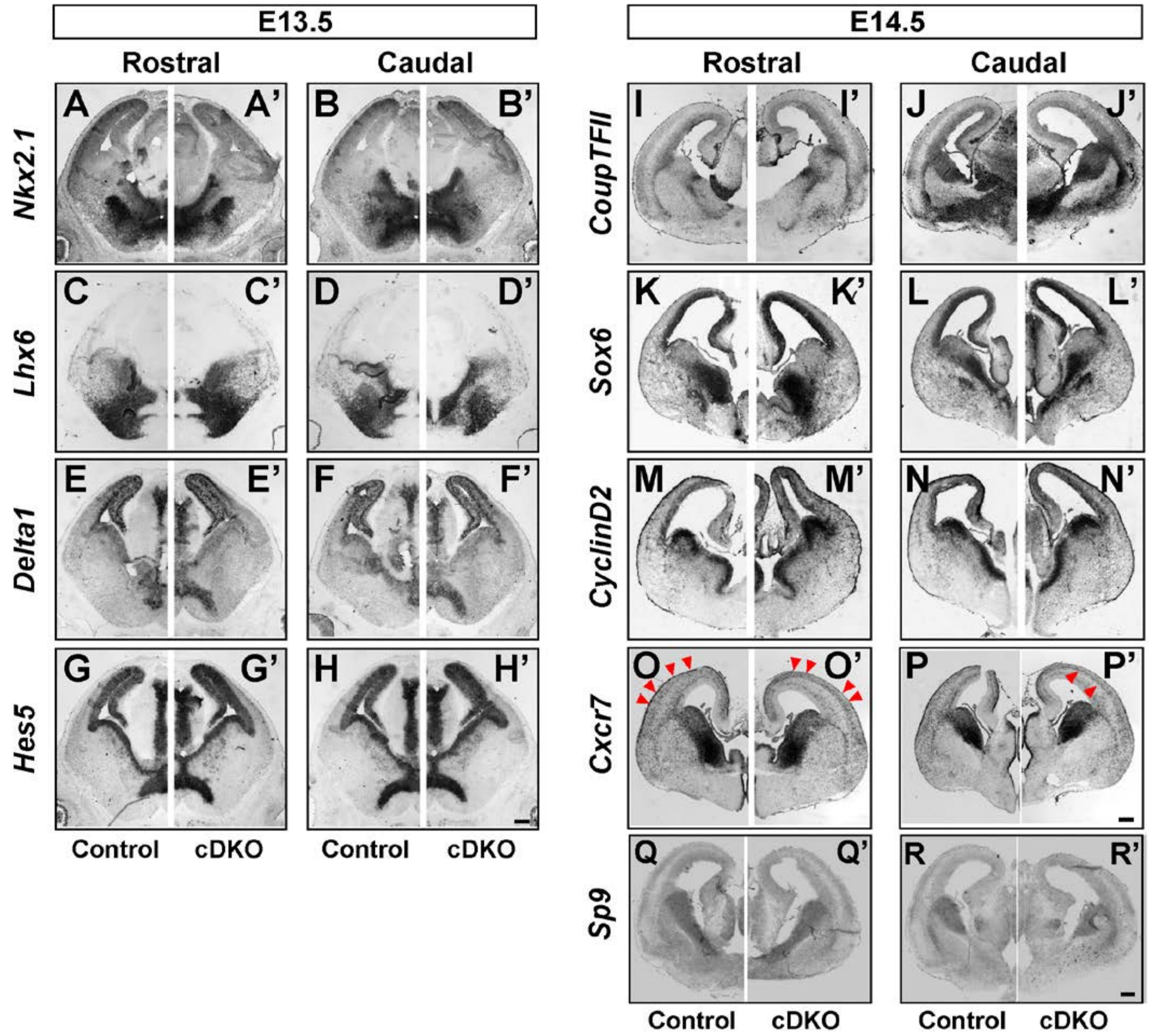

Figure S5. *In situ* hybridization for genes that regulate MGE and CIN development. *Related to Figure 2, Figure 3, Figure 4, and Figure 5.*

Rostral and caudal coronal hemisections through the telencephalon; *in situ* hybridization shows RNA expression of *Nkx2.1* **(A, A', B and B')**, *Lhx6* **(C, C', D and D')**, *Delta1* **(E, E', F and F')** and *Hes5* **(G, G', H and H')** at E13.5. *In situ* hybridizations showing expression of *CoupTFII* **(I, I', J and J')**, *Sox6* **(K, K', L and L')**, *CyclinD2* **(M, M', N and N')**, *Cxcr7* **(O, O', P and P')** and *Sp9* **(Q, Q', R and R')** at E14.5. In all panels, control hemispheres are on the left and cDKO hemispheres are on the right. Scale bars in **(H', P' and R')** = 250 μm.

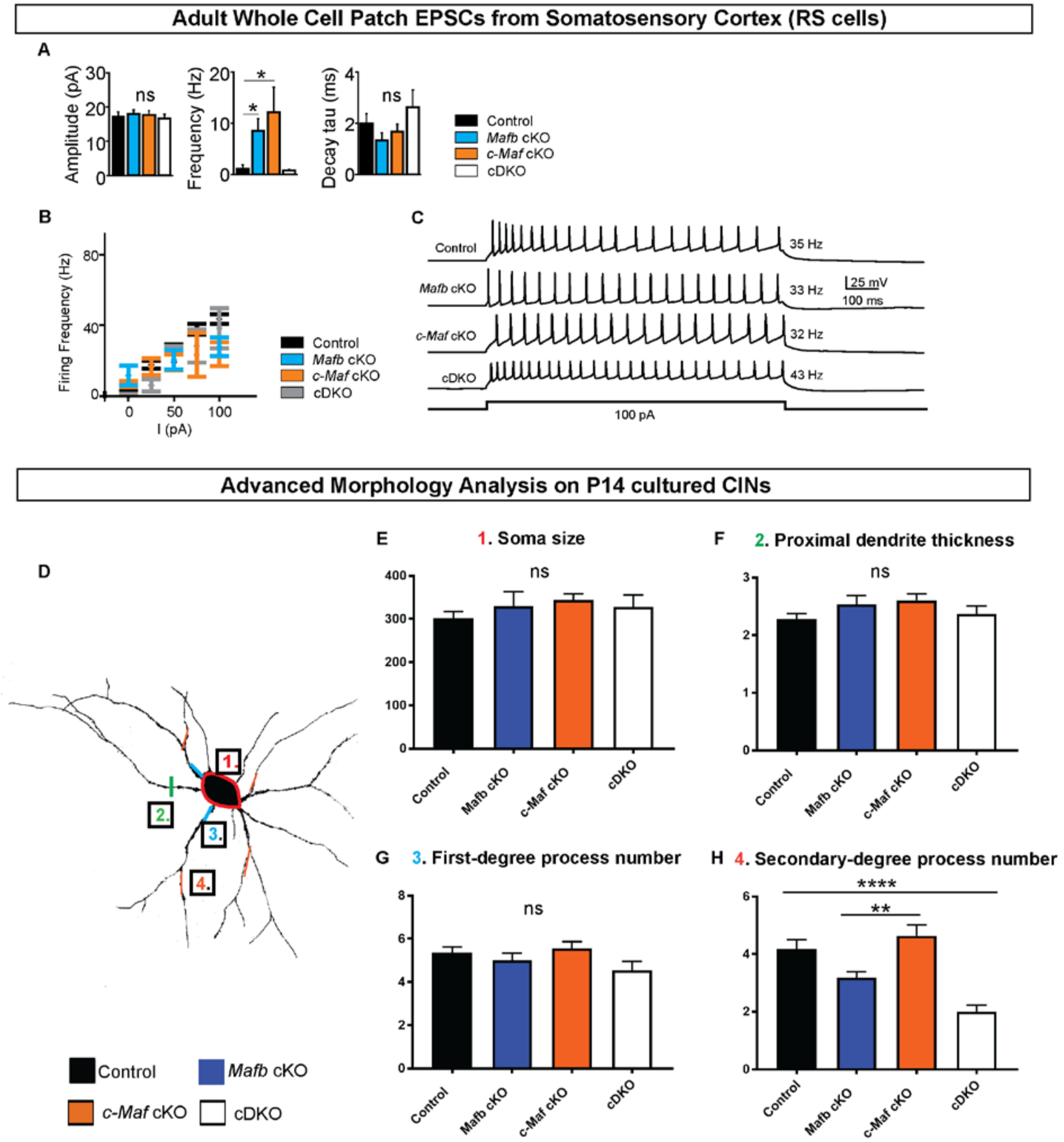

Figure S6. (A-C) Synaptic excitation and intrinsic excitability of RS CINs in control, *Mafb* cKO, *c-Maf* cKO and cDKO mice. (D-H) Neurite complexity analysis on CINs from control, *Mafb* cKO, *c-Maf* cKO and cDKO following 14 days of *in vitro* cortical culture. *Relate to Figure 6.*

(A) Quantification (mean ± SEM) of the amplitude, frequency and decay time constant of sEPSCs (Vhold = -70mV) in RS CINs. Note no change in amplitude or decay of sEPSCs and the increased frequency in *Mafb* and *c-Maf* cKOs compared with controls. *p < 0.05, One Way ANOVA Kruskal-Wallis. (Control: 7 cells; *Mafb* cKO:11 cells; *c-Maf* cKO: 6 cells; cDKO: 4 cells). **(B)** F-I curve: Plot of the mean action potential firing frequency as a function of current intensity injected in the RS cells. Overall, no significant change was observed between groups. **(C)** Representative traces from RS CINs for each genotype. **(D)** Schema depicting the regions that were quantified: soma size “1”, dendrite thickness “2”, primary neurite “3” and secondary neurite “4”. **(E)** Quantification (mean ± SEM) of soma size for 4 *Maf* genotypes. (Control: 15 cells; *Mafb* cKO: 12 cells; *c-Maf* cKO: 19 cells; cDKO: 15 cells) **(F)** Quantification (mean ± SEM) of proximal dendrite thickness. (Control: 43 neurites; *Mafb* cKO: 35 neurites; *c-Maf* cKO: 59 neurites; DKO: 38 neurites) **(G)** Quantification (mean ± SEM) of primary neurite numbers. (Control: 14 cells; *Mafb* cKO: 12 cells; *c-Maf* cKO: 18 cells; cDKO: 15 cells) **(H)** Quantification (mean ± SEM) of secondary neurite numbers. (Control: 21 cells; *Mafb* cKO: 17 cells; *c-Maf* cKO: 22 cells; cDKO: 24 cells). **p < 0.01, ****p < 0.0001; One Way ANOVA followed by Turkey’s multiple comparisons test.

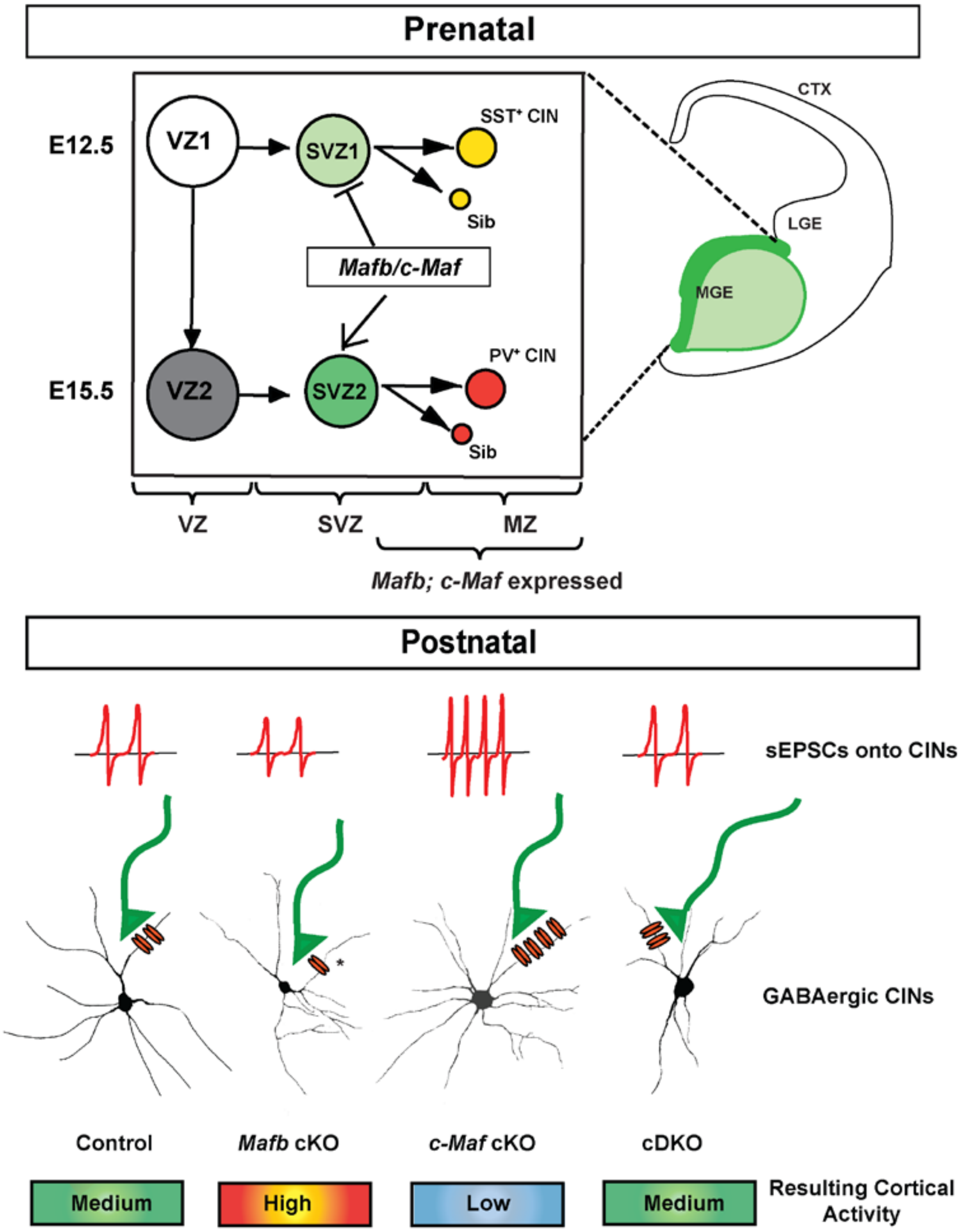

Figure S7. Models of *Mafb/c-Maf* function in MGE CIN cell type specification and dendritic/synaptic/function maturation. *Related to Figure 2, Figure 3, Figure 4, Figure 5, Figure 6 and Figure 7.*

Upper panel: Hypothesis for redundant prenatal roles for *Mafb* and *c-Maf* in CIN cell type specification. In the absence of both *Mafb* and *c-Maf* (cDKO) in the SVZ, there is excessive generation of SST^+ CINs. In the postnatal brain, after normalizing for the loss of CINs, there are decreased numbers of PV^+ CINs. We hypothesize that the reduction of PV^+ CINs may due in part to a role of *Mafb* and *c-Maf* in promoting PV^+ CIN identity in the SVZ of the MGE. Furthermore, we postulate that *Mafb* and *c-Maf* repress SST^+ CIN production/identity in the SVZ.

Lower panel: Opposing postnatal roles for *Mafb* and *c-Maf* in cortical excitability. In the *Mafb* and *c-Maf* cKO cortices, the numbers of MGE-derived CINs are roughly equal. However, loss of *Mafb* results in CINs that have (1) less post-synaptic densities (*= showing a decrease trend but didn't reach statistical significance), (2) receive less EPSCs and (3) a cortex that is hyper-responsive to a stimulus, while loss of *c-Maf* results in CINs that have (1) higher neurite complexity, (2) more post-synaptic densities, (3) receive greater EPSCs and (4) a cortex that is hypo-responsive to a stimulus. Notably, cDKO CINs have normal numbers of post-synaptic densities, receive a normal number of EPSCs and have a cortex that responded to a stimulus in a manner in between that of *Mafb* and *c-Maf* single cKOs.

Table S2. Cumulative cell counts of *Nkx2.1-Cre*-lineage cells in the neocortex and hippocampus

P35		Control	*Mafb* cKO	*c-Maf* cKO	*Maf* cDKO
	**Tissue**	**Total *Nkx2.1-Cre*-lineage cells/mm² ± SEM**			
	Hippocampus	266.1 ± 13.8	250.5 ± 5.2	257.5 ± 14.3	** 195.9 ± 8.2
	Neocortex	260.8 ± 10.1	** 194.5 ± 12.8	*** 176.7 ± 6.9	**** 87.2 ± 6.4
	Striatum	333.7 ± 9.8	* 298.4 ± 3.3	307.1 ± 6.3	*** 269.7 ± 7.0
**Marker**	**Tissue**	**Total *Nkx2.1-Cre*-lineage⁺/marker⁺ cells/mm² ± SEM**			
**PV**	Hippocampus	73.7 ± 3.1	68.1 ± 3.5	73.1 ± 8.3	*** 26.0 ± 3.4
	Neocortex	113.0 ± 9.5	* 76.3 ± 3.7 (32.5% decrease)	* 77.1 ± 6.3 (32% decrease)	*** 24.6 ± 6.5 (78.2% decrease)
	Striatum	73.4 ± 4.7	*** 38.1 ± 3.9	*** 44.0 ± 4.8	**** 7.6 ± 2.2
**SST**	Hippocampus	46.9 ± 9.5	50.7 ± 4.7	49.4 ± 9.9	26.1 ± 3.4
	Neocortex	101.5 ± 8.8	89.1 ± 7.4 (12% decrease)	* 72.0 ± 5.0 (29% decrease)	*** 31.0 ± 3.4 (69% decrease)
	Striatum	35.9 ± 2.5	32.4 ± 2.2	34.8 ± 2.2	34.2 ± 2.2
**Marker**	**Tissue**	**% *Nkx2.1-Cre*-lineage cells that express marker ± SEM**			
**PV**	Hippocampus	28.4 ± 1.6	28.7 ± 0.7	29.5 ± 2.3	*** 14.5 ± 1.4
	Neocortex	41.2 ± 1.7	39.7 ± 1.8	44.5 ± 0.8	*** 25.2 ± 3.4
	Striatum	22.7 ± 1.8	*** 12.6 ± 1.3	** 14.0 ± 1.3	*** 2.8 ± 0.8
**SST**	Hippocampus	17.9 ± 0.6	23.3 ± 2.3	21.8 ± 4.2	14.2 ± 2.2
	Neocortex	36.2 ± 1.8	41.1 ± 1.9	37.5 ± 0.5	38.1 ± 4.2
	Striatum	10.7 ± 0.9	11.1 ± 0.8	11.5 ± 0.7	12.6 ± 0.3

	Control	*Mafb* cKO	*c-Maf* cKO	*Maf* cDKO
**Tissue**	**Total *Nkx2.1-Cre*-lineage cells/mm² ± SEM**			
P7 Hippocampus	369.95 ± 8.16	* 258.88 ± 17.03	349.55 ± 20.1	398.86 ± 26.46
P16 Hippocampus	266.06 ± 13.76	250.48 ± 5.21	257.54 ± 14.31	* 195.86 ± 8.24

p* value * < 0.05, ** < 0.01, *** < 0.001, * < 0.0001 compared to control group**

Table S2. Cumulative cell counts of *Nkx2.1-Cre*-lineage cells in the neocortex and hippocampus. *Related to Figure 2, Figure 4 and Figure S3*

(Top) Quantification of the numbers of *Nkx2.1-Cre*⁺ cells from all genotypes at P35. **(Bottom)** Quantification of the numbers of *Nkx2.1-Cre*⁺ cells from all genotypes in the hippocampus at P7 and P16. Panels at the top show both proportion and cell density of tdTomato⁺ cells that express SST or PV in the somatosensory cortex and hippocampus. Panels at the bottom show the tdTomato⁺ cell density count in the whole hippocampus (including DG, CA1 and CA2/3). Data are expressed as the mean ± SEM. *p < 0.05, **p <0.01, ***p < 0.001, ****p < 0.0001.

Table S3. Passive and Active electric membrane properties of layer V CINs

Passive electric membrane properties						
**Cell Type**	**Genotype**	**Vm (mV)**	**Rin (MΩ)**	**τm (ms)**	**Cm (pF)**	**# of cells**
**FS**	Control	-69.4 ± 1.7 (27)	268.7 ± 33.7 (18)	18.1± 1.8 (17)	45.3 ± 4.5 (18)	27
	Mafb cKO	-67.6 ± 1.5 (26)	278.7 ± 25.8 (25)	21.4 ± 1.6 (24)	41.5 ± 2.9 (18)	26
	c-Maf cKO	-70.2 ± 1.1 (41)	252.3 ± 22.2 (38)	19.8 ± 1.1 (37)	36.9 ± 3.6 (37)	41
	cDKO	-71.3 ± 1.8 (8)	225.5 ± 50.4 (9)	19.9 ± 2.4 (9)	33.0 ± 6.3 (10)	8
	Statistics	ns	ns	ns	ns	
**RS**	Control	-70.1 ± 1.3 (16)	528.6 ± 34.9 (7)	34.0± 4.3 (7)	45.1 ± 3.8 (7)	16
	Mafb cKO	-62.4 ± 2.8 (11)	320.2 ± 43.3c(11)	23.2 ± 2.6 (11)	48.1 ± 4.8 (11)	11
	c-Maf cKO	-72.0 ± 1.5 (19)	570.5 ± 108.9 (8)	37.0 ± 8.0 (8)	32.0 ± 4.1 (11)	19
	cDKO	-72.0 ± 3.4 (6)	238.7 ± 46.8 (5)	24.6 ± 4.0 (5)	58.1 ± 9.3 (7)	6
	Statistics	p=0.01 (Control vs *Mafb* cKO) p= 0.003 (*Mafb* cKO vs *c-Maf* cKO)	p=0.004 (Control vs *Mafb* cKO) p=0.0005 (Control vs cDKO) p=0.03 (*Mafb* cKO vs *c-Maf* cKO) p=0.04 (*c-Maf* cKO vs cDKO)	p=0.036 (Control vs *Mafb* cKO)	p=0.04 (Control vs *c-Maf* cKO) p=0.018 (*Mafb* cKO vs *c-Maf* cKO) p=0.01 (*c-Maf* cKO vs cDKO)	

Active electric membrane properties						
**Cell Type**	**Genotype**	**AP threshold (mV)**	**AP amplitude (mV)**	**AP Full-duration (ms)**	**AP Half-duration (ms)**	**# of cells**
**FS**	Control	-55.8 ± 0.9	58.5 ± 2.3	1.4 ± 0.1	0.7 ± 0.05	19
	Mafb cKO	-57.9 ± 0.8	65.8 ± 2.3	1.6 ± 0.1	0.7 ± 0.03	24
	c-Maf cKO	-54.2 ± 0.7	60.1 ± 1.3	1.4 ± 0.1	0.6 ± 0.03	38
	cDKO	-54.97 ± 1.1	60.8 ± 2.0	1.5 ± 0.1	0.6 ± 0.04	9
	Statistics	p=0.001 (*Mafb* cKO vs *c-Maf* cKO) p=0.049 (*Mafb* cKO vs cDKO)	p=0.03 (Control vs *Mafb* cKO) p=0.025 (*Mafb* cKO vs *c-Maf* cKO)	p=0.04 (*Mafb* cKO vs *c-Maf* cKO)	p=0.02 (*Mafb* cKO vs *c-Maf* cKO) p=0.047 (*Mafb* cKO vs cDKO)	
**RS**	Control	-58.2 ± 0.8	59.7 ± 5.2	2.4 ± 0.3	1.1 ± 0.09	5
	Mafb cKO	-54.9 ± 1.2	56.1 ± 4.4	2.2 ± 0.3	0.9 ± 0.09	8
	c-Maf cKO	-53.9 ± 1.2	49.4 ± 2.7	2.3 ± 0.3	0.9 ± 0.1	8
	cDKO	55.7 ± 4.5	45.7 ± 7.3	2.1 ± 0.1	0.8 ± 0.04	3
	Statistics	p=0.02 (Control vs *c-Maf* cKO)	ns	ns	ns	

Table S3. Passive and active electric membrane properties of CINs. *Related to Figure 6 and Figure S6.*

Quantification of the numbers of passive **(Top)** and active **(Bottom)** electric membrane properties of fast-spiking (FS) and regular-spiking (RS) CINs in Control, *Mafb* cKO, *c-Maf* cKO and cDKO. Data are expressed as the mean ± SEM.

Table S4. Properties of spontaneous excitatory post-synaptic currents (sEPSCs) of layer V CINs for all groups and mEPSCs of layer V CINs for Control and *Mafb* cKO

sEPSCs					
**Cell Type**	**Genotype**	**Amplitude (pA)**	**Decay τ (ms)**	**Frequency (Hz)**	**# of cells**
**FS**	Control	23.9 ± 1.9	1.2 ± 0.2	8.4 ± 1.8	13
	Mafb cKO	17.4 ± 0.6	1.2 ± 0.1	6.8 ± 1.5	24
	c-Maf cKO	20.9 ± 0.8	1.0 ± 0.1	16.6 ± 3.2	33
	cDKO	23.2 ± 2.5	1.4 ± 0.2	9.4 ± 3.4	7
	Statistics	p=0.0004 (Control vs *Mafb* cKO) p=0.002 (*Mafb* cKO vs *c-Maf* cKO) p=0.002 (*Mafb* cKO vs cDKO)	p=0.03 (c-*Maf* cKO vs cDKO)	p=0.015 (*Mafb* cKO vs *c-Maf* cKO)	
**RS**	Control	17.4 ± 1.2	2.0 ± 0.4	1.2 ± 0.7	7
	Mafb cKO	18.1 ± 1.1	1.7 ± 0.3	8.6 ± 2.4	11
	c-Maf cKO	17.7 ± 1.3	1.4 ± 0.3	12.2 ± 4.8	6
	cDKO	16.7 ± 1.2	2.7 ± 0.7	0.8 ± 0.2	4
	Statistics	ns (p>0.1)	ns (p>0.1)	p=0.029 (Control vs *Mafb* cKO) p=0.03 (Control vs *c-Maf* cKO)	

mEPSCs				
**Measurement**	**Unit**	**Control**	***Mafb* cKO**	**Statistics**
**Frequency**	Hz	3.785 ± 0.7408	8.789 ± 2.748	Ns
**Amplitude**	pA	18.32 ± 0.963	12.54 ± 0.4074	P<0.0001
**Charge**	fC	32.56 ± 3.667	23.29 ± 3.299	P<0.01
**Tau Decay**	ms	1.588 ± 0.1679	1.973 ± 0.2371	ns
**Rise Time**	ms	0.2996 ± 0.009	0.326 ± 0.01389	ns
**Half-width**		1.996 ± 0.2647	2.252 ± 0.3733	ns
**Number of cells**		24	19	
**Number of mice**		2	2	

Table S4. Properties of spontaneous excitatory post-synaptic currents (sEPSCs) of layer V CINs for all groups and mEPSCs of layer V CINs for Control and *Mafb* cKO. *Related to Figure 6 and Figure S6***.**

(Top) Quantification of the numbers of properties of spontaneous excitatory post-synaptic currents (sEPSCs) of fast-spiking (FS) and regular-spiking (RS) CINs in Control, *Mafb* cKO, *c-Maf* cKO and cDKO. One Way ANOVA Kruskal-Wallis followed by Dunn's multiple comparisons test was used for the analysis. **(Bottom)** Quantification of the numbers of properties of mEPSCs of layer V CINs for Control and *Mafb* cKO. Mann-Whitney test was used for the analysis. Data are expressed as the mean ± SEM. *p < 0.05, **p <0.01, ***p < 0.001, ****p < 0.0001.

Table S5. Statistical analysis for local field potential measurement by layers

	From Figure 8D					
	Line length p values			Amplitude p values		
**Genotypes**	**Layer ii-iii**	**Layer iv**	**Layer v-vi**	**Layer ii-iii**	**Layer iv**	**Layer v-vi**
Control vs. *Mafb* cKO	< 0.0001	< 0.0001	< 0.0001	< 0.0001	< 0.0001	< 0.0001
Control vs. *c-Maf* cKO	0.006	0.02	< 0.0001	< 0.0001	< 0.0001	< 0.0001
Control vs. cDKO	0.0006	0.03	< 0.0001	< 0.0001	< 0.0001	< 0.0001
Mafb cKO vs. cDKO	< 0.0001	< 0.0001	< 0.0001	< 0.0001	< 0.0001	< 0.0001
Mafb cKO vs. *c-Maf* cKO	< 0.0001	< 0.0001	< 0.0001	< 0.0001	< 0.0001	< 0.0001
c-Maf cKO vs. cDKO	n.s.	0.0006	< 0.0001	< 0.0001	< 0.0001	< 0.0001

	From Figure 8E					
	Line length p values			Amplitude p values		
**Genotypes**	**Layer ii-iii**	**Layer iv**	**Layer v-vi**	**Layer ii-iii**	**Layer iv**	**Layer v-vi**
Control vs. *Mafb* cKO	< 0.0001	< 0.0001	< 0.0001	< 0.0001	< 0.0001	< 0.0001
Control vs. *c-Maf* cKO	n.s.	n.s.	0.02	n.s.	n.s.	n.s.
Control vs. cDKO	n.s.	n.s.	n.s.	n.s.	n.s.	n.s.
Mafb cKO vs. cDKO	< 0.0001	< 0.0001	n.s.	< 0.0001	0.001	< 0.0001
Mafb cKO vs. *c-Maf* cKO	< 0.0001	< 0.0001	0.03	< 0.0001	< 0.0001	0.01
c-Maf cKO vs. cDKO	n.s.	n.s.	n.s.	n.s.	n.s.	n.s.

Table S5. Statistical analysis for local field potential measurement by layers. *Related to Figure 7.*

Statistical analysis results for the local field potential/ current source density measurement by layers. Data presented here are p-values.
